# Diverging functional strategies but high sensitivity to an extreme drought in tropical dry forests

**DOI:** 10.1111/ele.13659

**Published:** 2020-12-14

**Authors:** Roy González‐M., Juan M. Posada, Carlos P. Carmona, Fabián Garzón, Viviana Salinas, Álvaro Idárraga‐Piedrahita, Camila Pizano, Andrés Avella, René López‐Camacho, Natalia Norden, Jhon Nieto, Sandra P. Medina, Gina M. Rodríguez‐M., Rebeca Franke‐Ante, Alba M. Torres, Rubén Jurado, Hermes Cuadros, Alejandro Castaño‐Naranjo, Hernando García, Beatriz Salgado‐Negret

**Affiliations:** ^1^ Programa Ciencias Básicas de la Biodiversidad Instituto de Investigación de Recursos Biológicos Alexander von Humboldt Cr. 1 # 16‐20 Bogotá Colombia; ^2^ Department of Biology Faculty of Natural Sciences Universidad del Rosario Cr. 24 # 63C‐69 Bogotá Colombia; ^3^ Institute of Ecology and Earth Sciences University of Tartu Lai 40 Tartu 51005 Estonia; ^4^ Fundación Jardín Botánico de Medellín, Herbario “Joaquín Antonio Uribe” (JAUM) Cll. 73 # 51D‐14 Medellín Colombia; ^5^ Departamento de Biología Facultad de Ciencias Naturales Universidad Icesi Cll. 18 # 122‐135 Pance Cali Colombia; ^6^ Facultad del Medio Ambiente y Recursos Naturales Universidad Distrital Francisco José de Caldas Cr. 5 Este # 15‐82 Bogotá Colombia; ^7^ Fundación Ecosistemas Secos de Colombia Cll. 5A Bogotá # 70C‐31 Colombia; ^8^ Dirección Territorial Caribe Parques Nacionales Naturales de Colombia Cll. 17 # 4‐06 Santa Marta Colombia; ^9^ Departamento de Biología Facultad de Ciencias Universidad de Valle Cll. 13 # 100‐00 Cali Colombia; ^10^ Asociación GAICA Cll. 11A # 32‐21 Pasto Colombia; ^11^ Programa de Biología Universidad del Atlántico Km. 7 vía Puerto Barranquilla Colombia; ^12^ Jardín Botánico del Valle del Cauca Juan María Céspedes ‐ INCIVA Cra. 27 #31‐47 Tuluá Colombia; ^13^ Departamento de Biología Universidad Nacional de Colombia Bogotá Colombia

**Keywords:** Biomass, demographic rates, hydraulic safety‐efficiency trade‐off, investment in tissues, trait probability density

## Abstract

Extreme drought events have negative effects on forest diversity and functioning. At the species level, however, these effects are still unclear, as species vary in their response to drought through specific functional trait combinations. We used long‐term demographic records of 21,821 trees and extensive databases of traits to understand the responses of 338 tropical dry forests tree species to ENSO_2015_, the driest event in decades in Northern South America. Functional differences between species were related to the hydraulic safety‐efficiency trade‐off, but unexpectedly, dominant species were characterised by high investment in leaf and wood tissues regardless of their leaf phenological habit. Despite broad functional trait combinations, tree mortality was more widespread in the functional space than tree growth, where less adapted species showed more negative net biomass balances. Our results suggest that if dry conditions increase in this ecosystem, ecological functionality and biomass gain would be reduced.

## Graphical abstract

Extreme drought events have negative effects on forest diversity and functioning. We used long‐term demographic records of 21,821 trees and extensive databases of traits to understand the responses of 338 tropical dry forests tree species to an extreme. Our results indicate that irrespective of the drought adaptations, most trees will be negatively affected under drier scenarios predicted for tropical dry forests.

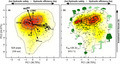

## Introduction

Tropical forest diversity and functioning are continuously threatened by a global increase in drought events (Allen *et al*., 2010, 2015; Nunes Garcia *et al*., 2018). Extreme droughts can increase tree mortality and significantly reduce ecosystem biomass gain due to low tree growth and recruitment (Condit *et al*., 1996; Slik, 2004; Allen *et al*., 2010; Maza‐Villalobos *et al*., 2013), even in forests considered to be historically water‐limited such as tropical dry forests (TDF; Allen *et al*., 2015; Powers *et al*., 2020). However, it is still not entirely clear how extreme drought events affect ecosystem processes, nor which mechanisms mediate species’ responses to drought (Allen *et al*., 2010). Trait‐based ecology provides a framework to understand how species respond to environmental filters, how they vary demographically, and, ultimately, what their fitness is (Pistón *et al*., 2019). Thus, studying the functional responses of species to extreme drought events, and how these translate into changes in biomass, can improve our ability to predict forests changes under future drier scenarios (Aubry‐Kientz *et al*., 2013; McDowell *et al*., 2018).

There is a general expectation that TDF tree species are adapted to cope with water limitation and should be more resistant to drought than species from mesic ecosystems (Dodd and Ryan, 2016). Studies have shown changes in traits in response to both longer and more frequent dry periods, such as a decrease in stature, and an increase in wood density (Esquivel‐Muelbert *et al*., 2019) and deciduousness (Fauset *et al*., 2012), suggesting that some traits provide an advantage under water constraints (Dodd and Ryan, 2016). Nevertheless, critical knowledge gaps remain regarding the viability of multiple trait combinations (Méndez‐Alonzo *et al*., 2012), trait relationships with demographic rates under droughts (Mendivelso *et al*., 2013; Allen *et al*., 2017b), or the role of traits in explaining species’ dominance (Prado‐Junior *et al*., 2016; Aguirre‐Gutiérrez *et al*., 2019). This lack of knowledge is especially acute given the difficulty of having simultaneous trait and demographic data for a large number of tree species during extreme droughts, information which will ultimately be essential to help us forecast how TDF may respond to future drier scenarios (Allen *et al*., 2010; Aguirre‐Gutiérrez *et al*., 2019).

**Figure 1:**
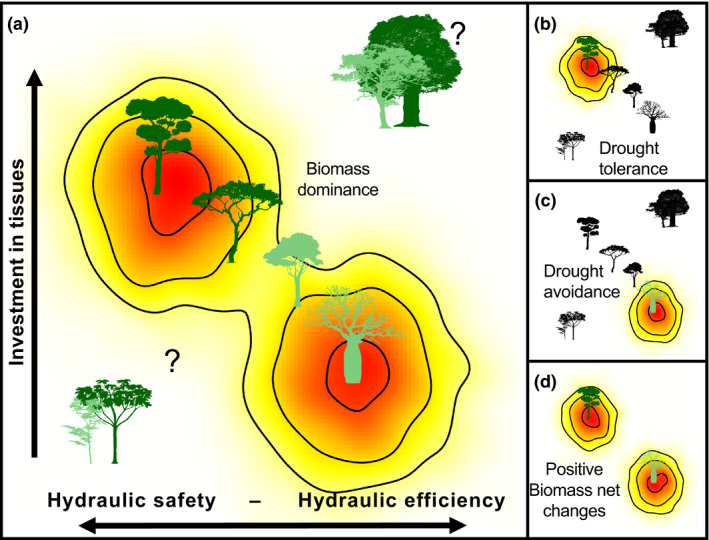
Schematic diagram representing hypotheses about the distribution of functional trait combinations across the correlated hydraulic safety‐efficiency and investment in tissues trade‐off axes (a, adapted from Méndez‐Alonzo *et al*., 2012). Within this trait space, dominant species (with higher biomass) are expected to be bounded by trade‐offs in trait combinations that favour drought tolerance (b) or drought avoidance (c). Therefore, positive biomass net changes (red areas in the continuum) can be expected for both strategies under an extreme drought (d). Here, both drought tolerance and drought avoidance are alternative optimal strategies for water‐constraints in TDF. Because species with other trait combinations are not expected to cope with drought conditions, they should not be present or be associated to low performance under extreme drought conditions. Dark green tree silhouettes represent evergreen species and light green tree silhouettes deciduous species.

Previous studies examining traits in TDF suggest that tree species are distributed along a continuum of functional traits related to a hydraulic safety‐efficiency trade‐off, which in turn should be related to the degree of investment in tissues (from ‘costly’ to ‘cheap’ tissues) (Fig. 1; Markesteijn *et al*., 2011a,b; Méndez‐Alonzo *et al*., 2012; Pineda‐García *et al*., 2015). On the one hand, species should have a high density of narrow xylem conduits, high carbon investment in thicker fibre cell walls, denser wood and leaves and high leaf retention time (Méndez‐Alonzo *et al*., 2012). These species with costly, but hydraulically safe tissues, are expected to be drought‐tolerant, exhibit low photosynthetic capacity and low growth rates due to a slow water transport, and a low mortality risk related to narrow and reinforced conduits support, more negative water potentials and enhanced mechanical stability (Poorter *et al*., 2008; Markesteijn *et al*., 2011a; Beeckman, 2016). On the other hand, species should have soft wood with larger xylem conduits and thin leaves with short retention time (Méndez‐Alonzo *et al*., 2012). These species are expected to have fast growth rates during the rainy seasons due to a high water transport capacity and photosynthetic rates (Santiago *et al*., 2004; Markesteijn *et al*., 2011a). However, they also have low hydraulic safety margins and may suffer higher mortality when water becomes limited (Méndez‐Alonzo *et al*., 2012; Pineda‐García *et al*., 2015).

Based on the above, we can expect drought‐tolerant species to be more dominant and less vulnerable to extreme droughts in TDF than species with ‘cheap’ but hydraulically efficient tissues (Fig. 1b) (Méndez‐Alonzo *et al*., 2012; Pineda‐García *et al*., 2015). Yet, the predominance of deciduous species in TDF (Pennington *et al*., 2009; Linares‐Palomino *et al*., 2011) suggests that lower leaf retention time in species that are hydraulically efficient is also a successful strategy to survive under high water constraints (Fig. 1c; Markesteijn *et al*., 2011a,b). By dropping their leaves, these drought‐avoidant species reduce hydraulic cavitation risks via lower transpiration rates and prevent drought‐induced mortality (McDowell *et al*., 2018). Accordingly, both drought‐tolerant and drought‐avoidance functional strategies should be successful in TDF (Sterck *et al*., 2011). The pervasive existence of these functional strategies in TDF (Méndez‐Alonzo *et al*., 2012) carries the idea that other trait combinations are unlikely to occur or, when present, they will perform poorly under strong water constraints (Fig. 1a–c; Ziemińska *et al*., 2015; Gleason *et al*., 2016). For instance, it has been demonstrated that high hydraulic conductivity and high wood density do not simultaneously occur in stems (Gleason *et al*., 2016), or that species cannot simultaneously grow stems that have high wood density, high fibre wall fractions and large fibre lumens (Ziemińska *et al*., 2015). Likewise, large‐leaves species with dense wood would have higher risks of cavitation in low rainfall ecosystems, as the result of major transpiration demands (Baraloto *et al*., 2010). However, the role of trade‐offs in hydraulic safety‐efficiency and tissues investment in determining species dominance in TDF or demographic responses to extreme droughts is still untested. Similarly, little is known about the existence of other trait combinations and their consequences on species performance in TDF.

Here, we used a network of 11 1‐ha permanent plots in TDF with a comprehensive data set of standing biomass and demography for 21,821 individual trees belonging to 338 species, and measurements of 15 leaf and hydraulic functional traits, to construct the functional trait space and relate it to species dominance and responses to the extreme ‘El Niño’ drought of 2015 in Northern South America (ENSO_2015_). We assessed three main questions: (1) Does the functional trait space reflects correlated trade‐offs axes in hydraulic safety‐efficiency and investment in tissues? (2) What are the dominant functional trait combinations of tree species? (3) How is the functional space related to demographic changes in biomass and net biomass balance after ENSO_2015_? Overall, we expected dominant species (high biomass) to exhibit combinations of functional traits that would fall along correlated trade‐off axes of hydraulic safety‐efficiency and investment in tissues (Fig. 1a). Yet, we also expected to find species with trait combinations outside these trade‐offs but with low dominance and poor performance after ENSO_2015_. Specifically, species with high hydraulic safety and ‘costly’ tissues (Fig. 1b, drought‐tolerant) or high hydraulic efficiency and ‘cheap’ tissues (Fig. 1c, drought‐avoidant), historically adapted to cope with water constraints, should experience lower mortality and higher growth rates under ENSO_2015_ compared to species with other functional trait combinations. These species would, in turn, have a high positive biomass net balance after ENSO_2015_ (Fig. 1d), whereas others will show a negative net biomass balance.

## Material and Methods

### Study area and censuses data

**Figure 2:**
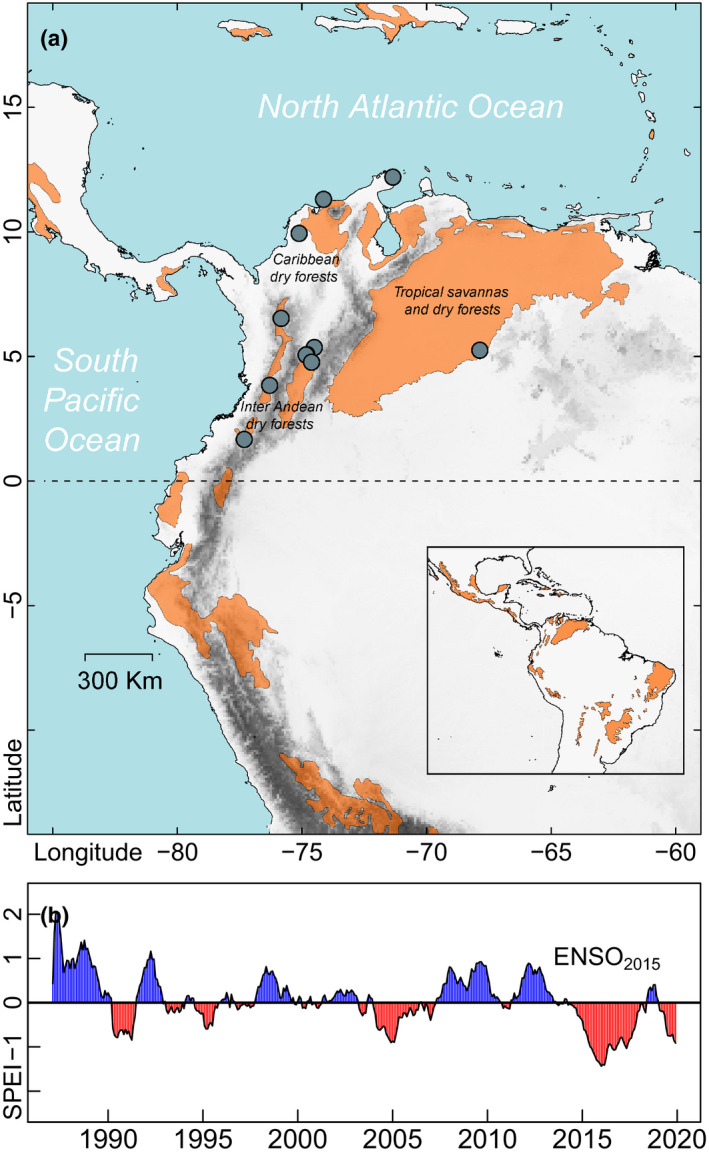
Geographical distribution and average inter‐annual drought regimes of the study sites. (a) Distribution of dry ecosystems in Northern South America (orange area, adapted from Pennington *et al*., 2018). Blue circles indicate the location of the 11 1‐ha permanent plots installed for monitoring mature forests across the region. (b) The Standardised Precipitation‐Evapotranspiration Index (SPEI; Vicente‐Serrano *et al*., 2012) was calculated based on long‐term data from weather stations near the plots (1980–2019). SPEI determines the magnitude and strength of drought conditions during the period of analysis, where negative values indicate the SPEI mean for drought periods (red colour) and positive values correspond to wet periods (blue colour). All plots experienced the extreme ENSO_2015_ (red area between 2015 and 2016). For extended details see Table S1 in supporting information

Between 2013 and 2014, we established 11 1‐ha permanent plots in TDF of Colombia, Northern South America (Fig. 2a). Plots were located in mature forests with floristic representativeness of the three main dry formations in the region (dry forest in the Caribbean, Inter Andean and Tropical Savannas regions; Fig. 2a and Table S1) without evidence of logging. Mean annual temperature varied between 23.4 and 28.3 ℃, mean annual precipitation between 517.0 and 2697.2 mm, and mean annual potential evapotranspiration between 1161 and 2067 mm. All sites experienced between one and two dry seasons (4–9 dry months) and soils had high proportions of sand (34.2–72.3%) and high aridity (0.8–3.4; see Table S1 for more details). Within each plot, all individual trees with a diameter at breast height ≥ 2.5 cm (DBH, censused at 1.3 m height) were tagged, and their DBH and height (m) were recorded (van Laar and Akça, 2007). Subsequently, 21,821 individual trees (26,132 stems) were resampled in all plots, and DBH of surviving trees and recruits (new individuals with DBH ≥ 2.5 cm) were measured between 2016 and 2017. Between censuses, all sites experienced one of the strongest drought events of the last 36 years, “El Niño” Southern Oscillation 2015–2016 (Fig. 2b, ENSO_2015_, Kogan and Guo, 2017). During this event, temperatures were almost 3°C above mean annual values and cumulative rainfall anomalies reached −250 mm (Anyamba *et al*., 2019), inducing intense water deficits in several areas of Neotropical dry forests (Fig. 2b, Table S1). ENSO_2015_ started in October 2014 with dry peaks between May 2015 and January 2016 and lasted until June 2016 (L’Heureux *et al*., 2017).

### Functional traits

We measured 15 functional traits in 1553 individual trees of 524 populations belonging to 338 species, where a population refers to all sampled individuals of the same species within a plot. We considered individual populations separately to account for local trait and biomass variations of species among plots. We measured four leaf traits and eleven wood traits, which characterise the hydraulic safety‐efficiency trade‐off as well as investment in tissues across a broad range of values (Table 1; Scholz *et al*., 2013; Salgado‐Negret *et al*., 2015). We collected traits for all tree species in each plot following an abundance‐weighted trait sampling scheme (Carmona *et al*., 2015). Accordingly, within each plot, we measured traits in 5–8 individuals for the most abundant species, 1–3 individuals for species with less than five individuals per plot, and one individual for species with only one individual per plot. For individual trees for which some traits had missing values (only 4% of sampled individuals, for a total of 0.98%), we imputed trait values using the R package “*missForest*” (missing value imputation for mixed‐type, Stekhoven and Bühlmann, 2012). We accounted for differences in the imputation process by including plots and species as predictors. Species with imputed individual‐trait values were strongly linearly correlated with their not‐imputed trait values (*P* < 0.001, Fig. S1).

**Table 1:** Description of the selected functional traits, trait functional dimensions, mean–ranges and global reference ranges

Trait (abbreviation)	Units	Description	Trait function (dimension)	Trait mean ± SD (${\bar{Q}}_{>0.1}$– ${\bar{Q}}_{<0.9}$)	Reference range	References
Fibre wall thickness (FWT)	µm	^a^Double wall between adjacent fibres ^b^Resistance of internal and external stresses ^c^Greater walls, higher hydraulic safety	Water exploitative safety (wood)	5.54 ± 1.52 (3.24–8.55)	4–12	Madsen and Gamstedt (2013); Scholz *et al*. (2013); Sorieul *et al*. (2016)
Hydraulically weighted diameter (d_h_)	µm	^a^Sum of circle conduits diameters *d* divided by the number of conduits *N* in a surface area ${\left(\sum \frac{d^{4}}{N}\right)}^{0.25}$ ^b^Conductance of conduits ^c^Larger weighted diameters, higher hydraulic efficiency	Water exploitative efficiency (wood)	58.64 ± 22.39 (31.23–105.74)	1–300	Scholz *et al*. (2013); Rosell *et al*. (2017)
Leaf area (LA)	mm^2^	^a^Projected area of a leaf ^b^Light interception, energy and water balance ^c^Larger LA, cheaper tissues and high water demands	Investment in tissues Water exploitative efficiency (leaves)	1.25 × 10^4^ ± 2.15 × 10^4^ (1.05 × 10^3^–5.77 × 10^4^)	1–>20×10^6^	Pérez‐Harguindeguy *et al*. (2013); Díaz *et al*. (2016)
Leaf dry matter content (LDMC)	mg g^−1^	^a^Dry mass per unit of lamina surface area ^b^Tissue investments and carbon‐gain strategies ^c^Higher LDMC, robust tissues	Investment in tissues (leaves)	379.38 ± 91.31 (209.46–533.64)	50–700	Pérez‐Harguindeguy *et al*. (2013); Díaz *et al*. (2016)
Leaf thickness (L_th_)	Mm	^a^Leaf mesophilic density (or thickness) ^b^Physical strength and leaf longevity ^c^Thicker leaves, higher tissue investments	Investment in tissues (leaves)	0.21 ± 0.06 (0.13–0.33)	0.11–0.74	Pérez‐Harguindeguy *et al*. (2013); Onoda *et al*. (2011)
Maximum vessel area (VA_max_)	µm^2^	^a^Average conduit surface area of the last VA percentile (>75, Q_3_–Q_4_) ^b^Hydraulically efficiency ^c^Greater conduits, higher water flows but higher conduits embolism risk	Water exploitative efficiency (wood)	2942.59 ± 2623.32 (589.12–8904.27)	7853–31415	IAWA *et al*. (2007); Scholz *et al*. (2013)
Pit area (PA)	µm^2^	^a^Pit aperture surface area ^b^Air–water interfaces for conduits ^c^Larger pits, higher water flows but higher conduits embolism risk	Water exploitative efficiency (wood)	19.68 ± 16.30 (4.37–55.15)	12–78	IAWA *et al*. (2007); Scholz *et al*. (2013)
Pit diameter aperture (DA_pit_)	µm	^a^Horizontal pit membrane diameter ^b^Embolism resistance inter‐conduits ^c^Smaller and denser pits, higher hydraulic safety	Water exploitative safety (wood)	2.90 ± 1.19 (1.38–5.38)	0.5–7	Scholz *et al*. (2013); Li *et al*. (2016); Helmling *et al*. (2018)
Specific leaf area (SLA)	mm^2^ mg^−1^	^a^Area of a fresh leaf divided by its oven‐dry mass ^b^Carbon capture and leaf longevity ^c^Higher SLA, lower tissue investments	Water exploitative efficiency (leaves)	15.39 ± 7.33 (7.24–32.22)	<1–300	Wright *et al*. (2004); Pérez‐Harguindeguy *et al*. (2013)
Vessel area (VA)	µm^2^	^a^Average conduit surface area ^b^Hydraulic conductivity ^c^Greater conduits, higher hydraulic efficiency but lower hydraulic safety	Water exploitative efficiency and safety (wood)	1676.93 ± 1484.11 (391.73–5094.72)	196–37600	Olson and Rosell (2013); Scholz *et al*. (2013)
Vessel density (VD)	vessels mm^−2^	^a^Number of conduits per cross‐sectional area ^b^Resistance to strength and vessel implosion ^c^Higher density, higher hydraulic safety	Water exploitative safety (wood)	71.71 ± 50.54 (15.22–181.83)	1–1000	Chave *et al*. (2009); Scholz *et al*. (2013); Jacobsen *et al*. (2005)
Wood density (WD)	g cm^3^	^a^Oven‐dry mass divided by saturated volume of the wood section ^b^Wood stability, aboveground biomass construction and carbon‐gain strategies ^c^Harder woods, lower water demands and higher tissue investments	Investment in tissues Water exploitative safety (wood)	0.63 ± 0.15 (0.32–0.84)	0.1–1.2	Chave *et al*. (2009); Pérez‐Harguindeguy *et al*. (2013)
Wood anhydrous density (WD_0_)	g cm^3^	^a^Oven‐dry mass divided by anhydrous volume of the wood section ^b^Wood stability ^c^Greater wood anhydrous densities, higher tissue investments	Investment in tissues (wood)	0.72 ± 0.17 (0.38–0.96)	0.1–1.5	Chave *et al*. (2009); Pérez‐Harguindeguy *et al*. (2013)
Water content at maximal capacity (WC_max_)	kg kg^−1^	^a^Free and fixed water capacity in cells. $\left[\left(1.5‐{\text{WD}}_{0}\right)\times 1.5{\text{WD}}_{0}\right]+{\text{WC}}_{\text{fsp}}$ (Water content at fibre saturation point); ${\text{WC}}_{\text{fsp}}=\frac{1}{\text{WD}}‐{\frac{1}{\text{WD}}}_{0}$ ^b^Shrinkage and swelling of xylem cells ^c^Higher water content, lower xylem mechanical resistance	Water exploitative efficiency (wood)	1.05 ± 0.61 (0.53–2.54)	0.2–5.0	Guevara (2001); Berry and Roderick (2005)
Xylem potential hydraulic conductivity (*K*_p_)	Kg m^−1^ s^−1^ MPa^−1^	^a^Theoretical specific xylem hydraulic conductivity per cross‐sectional area. $\frac{{\pi \rho }_{\omega }}{128\eta }\times \sum d_{h}^{4}\times \text{VD}$; $\rho_{\omega }$=998.2 kg m^‐3^; η = 1.002 × 10^–9^ MPa s^–1^; d_h_ and VD by m units ^b^Water exploitation abilities ^c^Higher potential conductivity, higher hydraulic efficiency	Water exploitative efficiency (wood)	25.09 ± 43.78 (2.25–113.72)	0.3–200	Chave *et al*. (2009); Poorter *et al*. (2010); Méndez‐Alonzo *et al*. (2012)

^a^ Trait‐based ecology definition and method of calculation.

^b^ Trait association to functions and mechanisms of a tree.

^c^ Trait association to hydraulic safety‐efficiency trade off of a tree.

### Standing biomass and biomass changes

To estimate biomass (tons, t) of each individual stem, we used the allometric formulas Type I and Type II for TDF from Alvarez et al. (2012), which consider DBH (cm), tree height (m) and stem wood density (g cm^−3^). Stem wood density was measured using the water displacement method and calculated as dry mass divided by fresh volume for 1–8 samples individual trees per species in each plot (Pérez‐Harguindeguy *et al*., 2013). Standing biomass of each species (t ha^−1^) was estimated as the sum of biomass of all its trees in each plot for the first census (t_0_). Biomass growth of survivors for each species (BG_S_, t ha^−1^ year^−1^) was estimated as the annual biomass increment produced by the growth of all trees surviving from t_0_ to the final census (t_fin_) in a plot. Biomass growth of recruits for each species (BG_R_, t ha^−1^ year^−1^) was estimated as the annual biomass increment obtained from all trees that attained at least 2.5 cm DBH in t_fin_ and that were not sampled in t_0_ in a plot. We considered that each new tree was recruited immediately after t_0_ and assumed that they had an initial DBH of 0 to avoid biomass overestimation (Talbot *et al*., 2014). Biomass mortality for each species (BM, t ha^−1^ year^−1^) was estimated as the biomass of dead trees between t_0_ and t_fin_. To correctly compare BM with BG_S_ and BG_R_, we calculated the biomass mortality of each tree as the biomass in t_0_ minus the biomass of the same tree calculated with a DBH of 2.5 cm (Talbot *et al*., 2014). Finally, we estimated net biomass change for each species (NBC, t ha^−1^ year^−1^) as net annual change in biomass during the time interval between t_0_ and t_fin_ (Prado‐Junior *et al*., 2016; Poorter *et al*., 2017), such that: NBC = BG_S_ + BG_R_ – BM.

### Statistical analyses

To characterise the functional trait space in TDF, we performed a Principal Component Analysis (PCA) with trait values at the individual level (i.e. each score in the PCA refers to an individual tree). We selected the two first PCA axes, which explained 61.32% of the variance, and performed a *varimax* rotation to improve the interpretability of the resulting two‐dimensional functional trait space. The rotation did not change the coordinate system of the initial PCA (ρ = 0.9, *P* < 0.001). Both axes of the functional space were defined as multidimensional traits for evaluating the trait probability density (TPD) of species, and their respective biomass dimensions (i.e. standing biomass, biomass demographics and biomass net changes), following the procedures in Carmona *et al*. (2016, 2019).

The TPD approach is based on the estimation of Gaussian kernel density functions (bivariate in this case) around each observation. Here, the TPD function of a given species represented the probabilities of observing different trait values (or combinations of them in the case of our two‐dimensional space) in those species, considering all sampled individuals (Carmona *et al*., 2016). For species with at least three individuals, the standard deviation (bandwidth) around each observation was selected using the unconstrained bandwidth estimation implemented in the R package *ks* (Chacón and Duong, 2018), as implemented in the *TPDs* function of the R package ‘*TPD*’ (Carmona *et al*., 2019). For species with less than or equal to two individuals within a plot, the standard deviation for each PCA axis and plot were predicted by regressing standard deviations against the mean value of species (considering all species within the plot). For extended details see Fig. S2.

To calculate the trait probability densities for each biomass dimension (TPD_C_), we combined all TPD of the individual species based on the sum of the probability functions, rescaled by the relative biomass dimension of each species (see Fig. S2). Then, to evaluate the amount of functional space occupied by each TPD_C_, we estimated the *Functional Richness* (F_Ric_) index suggested by Carmona *et al*. (2016, 2019). F_Ric_ refers to the sum of each biomass dimensions’ hypervolumes, considering their probability distributions for values above 0. We estimated differences in F_Ric_ between biomass dimensions (*e*.*g*. BG_S_ vs. BM) by calculating F_Ric_ 999 times, with half the species randomly selected each time. To evaluate dissimilarities on the occupancy of the functional space between biomass dimensions, we ran an *Overlap‐based functional dissimilarity* (β_O_) index, where values vary from 0 to 1 and indicate maximum dissimilarity between hypervolumes when β_O_ amounts to 1 (Carmona *et al*., 2019). We estimated if β_O_ was higher than expected by chance with a null model, in which TPDs scores were randomised and β_O_ was calculated 999 times (Traba *et al*., 2019). All statistical analyses were performed using R (v3.5.3; www.r‐project.org).

## Results

### Functional trait space and biomass dominance in TDF

**Figure 3:**
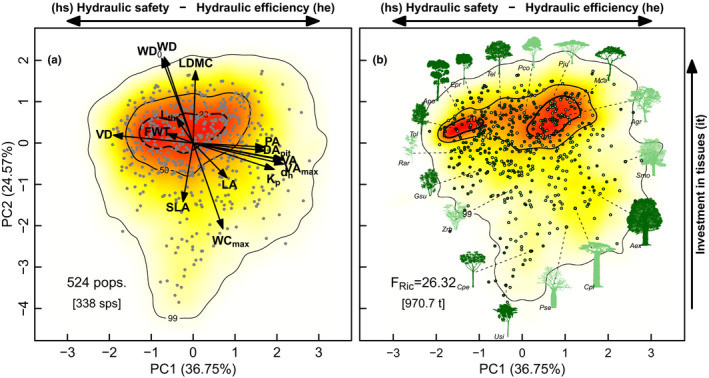
Trait probability densities (TPD) showing the functional trait combinations of species populations along an axis of hydraulic safety and efficiency trade‐off (hs‐he, PC1 36.75% explained variance) and an axis of investment in tissues (it, PC2 24.57% explained variance). (a) TPD where each species at each plot has an equivalent weight (grey points). (b) TPD where each species population is rescaled by its equivalent biomass in each plot (dark green points and tree silhouettes represent evergreen species and light green points and tree silhouettes deciduous species). Functional traits: Fibre wall thickness (FWT, µm), hydraulically weighted diameter (d_h_, µm), leaf area (LA, mm^2^), leaf dry matter content (LDMC, mg g^−1^), leaf thickness (L_th_, mm), maximum vessel area (VA_max_, µm^2^), pit area (PA, µm^2^), pit diameter aperture (DA_pit_, µm), specific leaf area (SLA, mm^2^ mg^−1^), vessel area (VA, µm^2^), vessel density (VD, vessels mm^−2^), wood density (WD, g cm^3^), wood anhydrous density (WD_0_, g cm^3^), water content at maximum capacity (WC_max_, kg kg^−1^), and xylem potential hydraulic conductivity (K_s_, kg m^−1^ s^−1^ MPa^−1^). Functional Richness (F_Ric_). Examples of species with different functional trait combinations in TDF*: Anacardium excelsum (Aex), Aspidosperma polyneuron (Apo), Astronium graveolens (Agr), Cavanillesia platanifolia (Cpl), Cecropia peltate (Cpe), Eugenia procera (Epr), Gustavia superba (Gsu), Machaerium capote (Mca), Pradosia colombiana (Pco), Prosopis juliflora (Pju), Pseudobombax septenatum (Pse), Randia armata (Rar), Spondias mombin (Smo), Trichilia oligofoliolata (Tol), Trichilia elegans (Tel), Urera simplex (Usi), Zanthoxylum rhoifolium (Zrh)*.

The functional trait space of 524 populations, belonging to 338 tree species, is summarised in the first two dimensions of the PCA (Fig. 3a). The first PCA axis (36.75% of explained variance) reflected the hydraulic safety‐efficiency trade‐off. The high safety side was characterised by a high density of narrow vessels with high fibre wall thickness, whereas the hydraulic‐efficiency side had wide vessels and pits with high xylem potential hydraulic conductivity. The second PCA axis (24.57% of explained variance) reflected differences of investment in tissues, where negative values were related to large leaves with high SLA, and high content of water at maximal capacity (‘cheap’ tissues), whereas positive values corresponded to high LDMC and high wood density (‘costly’ tissues). The 50% probability threshold in the TPD, which represents the functional dominance of species (TPD_<50%_; Fig. 3a), showed that more than half of the populations (288) occurred along the hydraulic safety‐efficiency trade‐off axis but were restricted to the side of high investment in tissues. Surprisingly, however, many populations had combinations of traits outside these trade‐off axes (TPD_50‐99%_ = 236; Fig. 3a). When TPD was rescaled by standing biomass, the TPD_<50%_ was narrower and included 220 populations, which accounted for 61% of total biomass (592.8 t, Fig. 3b). Populations with costly and hydraulically safe tissues and costly and hydraulically efficient tissues accounted for 37.4% of total biomass (TPD_<20%_= 76 populations with 362.9 t, Fig. 3b), whereas populations with other trait combinations were widespread but with low biomass across the functional space (Fig. 3b). For instance species with dominant trait combinations such as *Trichilia oligofoliolata* (costly tissue and hydraulically safe evergreen species) or *Astronium graveolens* (costly and hydraulically efficient deciduous species) reached to 53.6 and 21.5 t ha^−1^ respectively. In contrast, species with other trait combinations and low investment in tissues such as *Urera simplex* (an evergreen species with intermediate hydraulically efficiency) or *Pseudobombax septenatum* (the typical deciduous and hydraulic efficiency species) only reached 0.02 and 4.7 t ha^−1^ respectively (Fig. 3b, Table S2).

### TDF functional and biomass changes after ENSO_2015_

**Figure 4:**
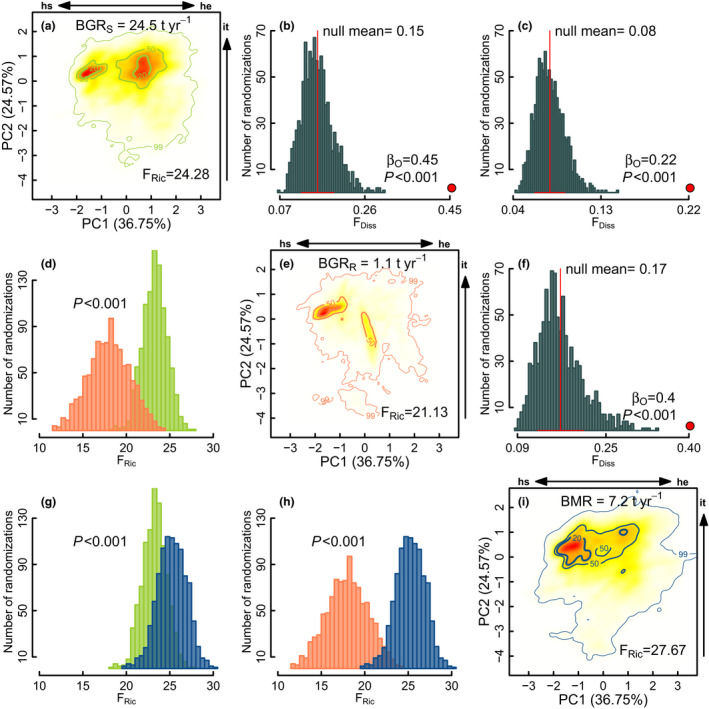
Trait probability densities (TPD) showing the functional trait combinations of species populations rescaled by biomass growth of survivors (a, BG_S_ in green colour), biomass growth of recruits (e, BG_R_ in orange colours) and biomass mortality (i, BMR in blue colour). Null models for Functional Dissimilarity (F_Diss_, b, c and f) between biomass growth and mortality TPD’s. Significant β_O_ (*P* < 0.001) indicates that dissimilarity between paired TPD demographic dimensions is greater than the expected by chance (999 randomisations). Functional Richness (F_Ric_, d, g and h) between biomass growth and mortality TPD’s (b, c and f). Significant differences between the paired frequency distributions indicate different F_Ric_ of the contrasted TPD’s demographic dimensions (*P* < 0.001, 999 randomisations). Hydraulic safety (hs), hydraulic efficiency (he), investments in tissues (ti).

For the entire set of 11 1‐ha permanent plots biomass growth of surviving trees was 24.5 t year^−1^ (2.23 t ha^−1^ year^−1^ ± 0.67), biomass of recruiting trees was 1.10 t year^−1^ (0.10 t ha^−1^ year^−1^ ± 0.11) and biomass mortality was 7.2 t year^−1^ (0.65 t ha^−1^ year^−1^ ± 0.35; Table S1). We found a high dissimilarity between functional trait spaces for all demographic dimensions at all probability thresholds (*P* < 0.001, Fig. 4 b, c, f, Fig. S3). The two dominant pre‐ENSO_2015_ functional trait combinations, hydraulically safe with high investment and hydraulically efficient with high investment, showed the largest post‐ENSO_2015_ biomass growth of surviving trees (TPD_20%_= 50 populations, 10.4 t year^−1^ [42% total biomass] and TPD_50%_= 167 populations, 14.3 t year^−1^ [58%]; Fig. 4a). In contrast, the largest biomass for recruited trees was restricted to species with combinations of costly and hydraulically safe traits or ‘cheap’ and hydraulically efficient traits (TPD_50%_= 27 species, 0.64 t year^−1^ [57%]; Fig. 4e). Species with low investment in tissues and high hydraulic efficiency were mainly deciduous (71%, *e*.*g*. *Zanthoxylum lenticulare = *0.17 t ha^−1^ year^−1^). The highest biomass loss due to mortality was mainly ascribed to species with costly but hydraulically safe tissues (TPD_20%_= 142 populations, 4.61 t year^−1^ [64%]; Fig. 4i), and a second group of functional trait combinations was related to species with costly and hydraulically efficient tissues (TPD_20‐50%_= 97 populations, 2.88 t year^−1^ [40%]; Fig. 4i). It is important to highlight that biomass loss by mortality covered a broader proportion of the functional space than other demographic dimensions (F_Ric_ = 27.67, *P* < 0.001, Fig. 1 d, g, h). These results were consistent across all tested probability thresholds (Fig. S4).

**Figure 5:**
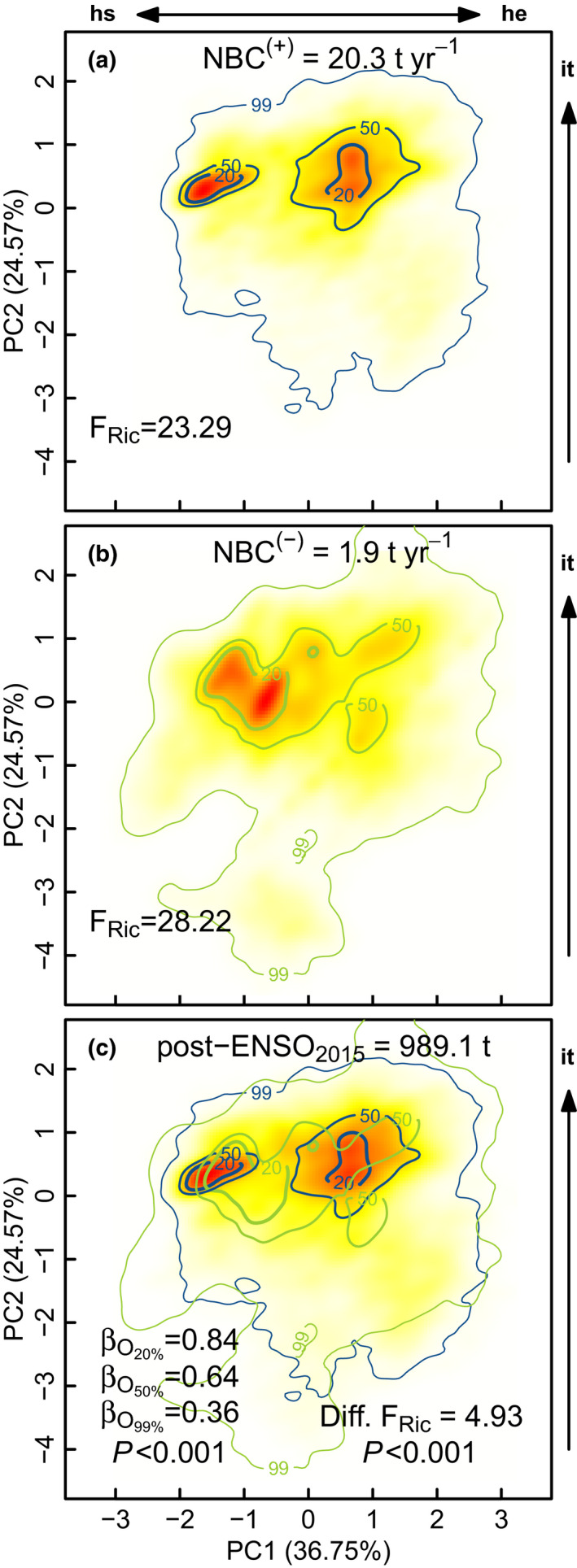
Trait probability densities (TPD) showing the functional space of trait combinations for species populations rescaled by positive net biomass changes (a, NBC^(+)^ in blue colours), by negative net biomass changes (b, NBC^(–)^ in green colours) and by standing biomass after ENSO_2015_ (c). F_Ric_ refers to functional richness and F_Diss_ to functional dissimilarity. Significant β_O_ (*P* < 0.001) indicate that functional dissimilarity between positive and negative net biomass change TPD’s is greater than expected by chance (999 randomisations). Differences in functional richness (Diff. F_Ric_, *P* < 0.001) indicate that negative net biomass changes TPD’s had a higher F_Ric_ than positive net biomass changes TPD’s (999 randomisations). Hydraulic safety (hs), hydraulic efficiency (he), investments in tissues (ti)

Net biomass change of all plots was 18.4 t year^−1^ (1.68 t ha^−1^ year^−1^ ± 0.56), varying between 0.82 and 2.50 t ha^−1^ year^−1^ per plot (Table S1). Positive changes in net biomass reached 20.3 t year^−1^ (1.84 t ha^−1^ year^−1^ ± 0.61) and were restricted to the dominant functional trait combinations (Fig. 5a, TPD_<50%_). Negative changes in net biomass attained 1.9 t year^−1^ (0.17 t ha^−1^ year^−1^ ± 0.11) and included species with costly hydraulically safe tissues, but also ‘intermediate’ species with traits between both ends of the hydraulic safety and efficiency trade‐off and with intermediate investment in tissues (Fig. 5b, TPD_<50%_). Functional dissimilarities between positive and negative net biomass changes (β_O_) were significant at all probability thresholds (*P* < 0.001; Fig. 5c). Negative biomass changes occupied a higher fraction of the functional space than positive biomass changes for all probability thresholds (Diff. F_Ric_ = 4.93, *P* < 0.001; Fig. 5c). Notably, the total biomass of all 1‐ha plots after ENSO_2015_ was 989.1 t, but the 50% threshold probability showed a clear divergence of both dominant functional trait combinations that were not detected for the initial biomass (see Fig. 3b and Fig. 5c).

## Discussion

Tropical dry forests (TDF) experience frequent water limitations as the result of annual rainfall seasonality (Linares‐Palomino *et al*., 2011) and inter‐annual extreme droughts (Allen *et al*., 2010). In response to these conditions, species have evolved a particular suite of functional traits (Pennington *et al*., 2009; Aguirre‐Gutiérrez *et al*., 2019), with important consequences for ecosystem functioning. To understand the functional responses of TDF tree species to extreme drought events, we assessed the functional trait space of a large number of tree species and evaluated if particular suites of trait determined differences in species standing biomass and demographic biomass changes to one of the driest events in decades (ENSO_2015_). Our results showed that: (1) TDF tree species are distributed across a broad functional trait space associated with trade‐off axes of hydraulic safety‐efficiency and investment in tissues. (2) Biomass‐dominant species were located along the hydraulic safety‐efficient trade‐off axis but only at the high investment tissues side. Yet, almost half of the species had other trait combinations but with low biomass. (3) Biomass loss by mortality covered a broader functional trait space than biomass growth, but net biomass losses were more strongly experienced by species with low investment in tissues and hydraulic traits intermediate between safety and efficiency.

### The hydraulic safety‐efficiency trade‐off and costly tissues govern species dominance in TDF

As expected, TDF tree species were broadly distributed along the hydraulic safety‐efficiency trade‐off axis (Fig. 3). We found a first large group of species with high standing biomass in the functional space combining hydraulically safe traits (Fig. 3b), such as narrow vessels and pit areas and high vessel density (Onoda *et al*., 2011; Markesteijn *et al*., 2011b; Méndez‐Alonzo *et al*., 2012). A second dominant group was associated with traits related to high water transport efficiency (Fig. 2a) such as wide vessels, large pit areas and high xylem potential hydraulic conductivity (Sobrado, 1997; Pineda‐García *et al*., 2015). However, and contrary to our expectations, this trade‐off axis was not related to a parallel trade‐off axis of tissue investments since both groups of dominant species were found to have dense leaves and stems (Fig. 2). This unexpected result disagrees with previous studies that have suggested that species with high hydraulic‐efficiency, traditionally associated with deciduousness, have low investment in tissues but high nutrient concentration which would enable them to maximise growth rates during their short growing season (Brodribb *et al*., 2010; Markesteijn *et al*., 2011a,b; Méndez‐Alonzo *et al*., 2012). In contrast, our results suggest that not investing in expensive tissues has negative consequences on biomass dominance, irrespective of leaf habit (Fig. 5c). Despite this result being novel, it is consistent with physiological mechanisms that allow species to face drought. For instance high wood density, which may result from a different combination of tissue and cell distributions such as high investments in fibre wall thickness (Ziemińska *et al*., 2015) or an increasing abundance of fibre (Jacobsen *et al*., 2007), can protect vessels from implosion when water shortage creates strong negative xylem potentials (Hacke *et al*., 2001; Pratt *et al*., 2007). Likewise, thick and dense leaves may be more resistant to drought because living cells have rigid walls preventing cell collapse caused by negative turgor pressures developing under substantial water loss (Salleo and Nakdini, 2000). Additionally, these leaves have smaller transpiring surfaces, hence reducing wilting and water requirements (Niinemets, 2001; Poorter *et al*., 2009). However, it is important to acknowledge that investment in costly tissues may also respond to other factors such as protection against herbivores and against other physical hazards (Turner, 1994; Cunningham *et al*., 1999). For instance *Aspidosperma polyneuron*, a dominant species with dense leaves, has showed lower leaf area removed by herbivory than *Sapium glandulosum*, which have low dominance and thin tissues (Silva *et al*., 2015; Table S2). Moreover, dense tissues in deciduous species may retard leaf loss during the dry seasons increasing the carbon gain window, or reduce cavitation risk during unexpected dry conditions in the rainy seasons (Powers and Tiffin, 2010; Lopezaraiza‐Mikel et al. 2013).

The fact that investment in expensive tissues relates to high standing biomass in TDF opens new questions about the mechanisms that may mediate the dominance patterns of species under future climatic scenarios not only in this ecosystem historically adapted to drought, but also in others where changes in rainfall regimens may be more dramatic for species and functional composition (*e.g*., moist and rain forests; Esquivel‐Muelbert *et al*., 2017, 2019). For example if extreme droughts become more frequent and intense in the tropics (Allen *et al*., 2010, 2015), and building dense tissues is energetically expensive and time‐consuming (Chave *et al*., 2009; Osnas et al. 2013), how will the functional space be modified in tropical forests in the future? Answering this question requires models that integrate not only species losses but also changes in the trait composition (Lawlor and Tezara, 2009; McDowell *et al*., 2018), where linking leaf, wood and hydraulic traits to biomass may help anticipate shifts in forests functioning facing climatic change (Esquivel‐Muelbert *et al*., 2019; Aguirre‐Gutiérrez *et al*., 2020).

### Biomass demographic changes in TDF following the ENSO_2015_

After ENSO_2015_ the functional space of biomass loss by mortality was significantly broader than the space of growth and recruitment (Fig. 4), and a broader range of functional trait combinations were associated with negative than with positive biomass changes (Fig. 5). These findings support the idea that under extreme droughts, tree mortality is more widespread in the functional space than growth, and that mortality is an important driver of functional composition and forests functioning (Allen *et al*., 2010; Fauset *et al*., 2012; Aguirre‐Gutiérrez *et al*., 2019; Esquivel‐Muelbert *et al*., 2019). Demographic rates were strongly shaped by the hydraulic safety‐efficiency axis and by high investment in tissues (Fig. 4). The higher biomass gained by survivors’ growth was restricted to species with costly, but hydraulically safe tissues or costly and hydraulically efficient tissues, which is consistent with the prediction that species having dominant functional trait combinations, and that are historically adapted to drought conditions, would perform better under water constraints. Our results also demonstrate that irrespective of the hydraulic designs or leaf habits, investment in expensive leaf and wood tissues allow species to grow under extreme droughts. This result may be related to the fact that building expensive tissues implies more biomass per volume fraction, where despite the expected low growth rates for the costly and hydraulically safety tissue species, they can pack high carbon stocks at constant growth rates during long periods (Poorter *et al*., 2017). Likewise, costly and hydraulically‐efficient tissue species have important carbon gains during the reduced growing season when they not only invest in performance but also protect their structures from coping with water‐constraints (Somavilla *et al*., 2014).

Interestingly, the biomass of recruited trees was the only demographic dimension following the expected coordination between the hydraulic safety‐efficiency trade‐off axis and the ‘costly’ to ‘cheap’ investment in tissues axis. Markesteijn *et al*. (2011a) found that pioneer and deciduous tree species generally combine high hydraulic conductivity with low wood density, which favours short‐term gain in biomass at the expense of long‐term survival. This strategy prioritises fast height growth, to rapidly reach the canopy after gap formation. This same explanation may apply in our study if we take into account that many of the recruited species had hydraulically efficient stems, a deciduous leaf habit, high specific leaf area and high water content at maximum capacity, which are all traits associated with fast growth (Wright *et al*., 2004; Poorter *et al*., 2008).

The broader functional space associated with tree mortality, in comparison to biomass gain by growth, suggests that most TDF species are sensitive to extreme droughts. Additionally, we observed that high mortality was concentrated in species with costly and hydraulically safe tissues, followed, to a lesser extent, by species with costly and hydraulically‐efficient tissues (Fig. 4i). This unexpected result contrasts with previous studies suggesting that ‘conservative’ traits associated with high hydraulic‐safety should be positively related to survival rates in TDF (Prado‐Junior *et al*., 2016; Powers *et al*., 2020). These conservative traits are probably adaptive under average drought conditions, but extreme droughts could surpass the high safety margins in these species and cause mortality. However, it is important to note that although biomass losses by mortality and functional space reduction were evident in this study as the result of the ENSO_2015_, long‐term monitoring is necessary to determine if under ‘normal’ climatic conditions, TDF recover their pre‐ENSO_2015_ biomass and functionality, or if, on the contrary, TDF species have widespread mortality risks across the whole hydraulic safety‐efficiency functional space under these particular dry events (Powers *et al*., 2020).

### The sensitivity of TDF to future drier scenarios

Future drier scenarios are expected to change the functional space and functioning of TDF (Allen *et al*., 2010, 2015). However, the strength and direction of these changes are still unclear because of the absence of studies on the functional sensitivity of species to extreme droughts, and the absence of long monitoring trait‐demographic data. Recently, it was shown that ENSO_2015_ caused high mortality of TDF species with low hydraulic safety margins (Powers *et al*., 2020). Our results suggest that, irrespective of their hydraulic designs, species with low investment in tissues were strongly sensitive to ENSO_2015_, resulting in important negative net biomass balances. In a broader context, biomass net balance after ENSO_2015_ was over two times lower than balances for a rainy period. For instance between 2009 and 2011 (wet period for TDF in Northern South America, SPEI> 1; Fig. 2b), El Vinculo, one of our study sites, had a net biomass gain of 3.4 t ha^−1^ year^−1^ (tress with DBH > 5 cm; Torres *et al*., 2012), whereas after ENSO_2015_ it only reached 1.73 t ha^−1^ year^−1^ (trees with DBH > 2.5 cm). This result, together with the narrower functional space of positive biomass gain than that of negative biomass gains (Fig. 5c), suggests that both functional diversity and biomass productivity could decrease in future drier scenarios. Further studies should model how future scenarios of changes in rainfall regimes may impact forest biomass dynamics (Allen *et al*., 2017a), but also consider if the functional trait space will become narrower due to these events.

## Authorship

RG‐M, HG and BS‐N planned and coordinated data collection; RG‐M, AI‐P, BS‐N, AMT, CP, GMR, RJ, RL‐C, SPM, HC, AC‐N and JN carried out the fieldwork; VS, FG, SPM and RG‐M performed the traits measurements; RG‐M, JMP, CPC and BS‐N developed the manuscript idea; RG‐M and CPC performed the statistical analysis; RG‐M, CPC, JMP, AA, NN, CP and BS‐N interpreted the results; RG‐M wrote the manuscript and all authors contributed to revisions.

### Peer Review

The peer review history for this article is available at https://publons.com/publon/10.1111/ele.13659.

## Supporting information

Supplementary Material

## Data Availability

The processed data that support the findings of this study are available in Dryad Digital Repository, https://doi.org/10.5061/dryad.ns1rn8pr8

## References

[ele13659-bib-0001] Aguirre‐Gutiérrez, J. , Malhi, Y. , Lewis, S.L. , Fauset, S. , Adu‐Bredu, S. , Affum‐Baffoe, K. *et al*. (2020). Long‐term droughts may drive drier tropical forests towards increased functional, taxonomic and phylogenetic homogeneity. Nat. Commun., 11, 1–10. 32620761 10.1038/s41467-020-16973-4 PMC7335099

[ele13659-bib-0002] Aguirre‐Gutiérrez, J. , Oliveras, I. , Rifai, S. , Fauset, S. , Adu‐Bredu, S. , Affum‐Baffoe, K. *et al*. (2019). Drier tropical forests are susceptible to functional changes in response to a long‐term drought. Ecol. Lett., 22, 855–865. 30828955 10.1111/ele.13243

[ele13659-bib-0003] Allen, C.D. , Breshears, D.D. & McDowell, N.G. (2015). On underestimation of global vulnerability to tree mortality and forest die‐off from hotter drought in the Anthropocene. Ecosphere, 6, 1–55.

[ele13659-bib-0004] Allen, C.D. , Macalady, A.K. , Chenchouni, H. , Bachelet, D. , McDowell, N. , Vennetier, M. *et al*. (2010). A global overview of drought and heat‐induced tree mortality reveals emerging climate change risks for forests. For. Ecol. Manage., 259, 660–684.

[ele13659-bib-0005] Allen, K. , Dupuy, J.M. , Gei, M.G. , Hulshof, C. , Medvigy, D. , Pizano, C. *et al*. (2017a). Will seasonally dry tropical forests be sensitive or resistant to future changes in rainfall regimes? Environ. Res. Lett., 12, 023001.

[ele13659-bib-0006] Allen, W.L. , Street, S.E. & Capellini, I. (2017b). Fast life history traits promote invasion success in amphibians and reptiles. Ecol. Lett., 20, 222–230. 28052550 10.1111/ele.12728 PMC6849728

[ele13659-bib-0007] Álvarez, E. , Duque, A. , Saldarriaga, J. , Cabrera, K. , de las Salas, G. , del Valle, I. *et al*. (2012). Tree above‐ground biomass allometries for carbon stocks estimation in the natural forests of Colombia. For. Ecol. Manage., 267, 297–308.

[ele13659-bib-0008] Anyamba, A. , Chretien, J.P. , Britch, S.C. , Soebiyanto, R.P. , Small, J.L. , Jepsen, R. *et al*. (2019). Global disease outbreaks associated with the 2015–2016 El Niño event. Sci. Rep., 9, 1–14. 30760757 10.1038/s41598-018-38034-z PMC6374399

[ele13659-bib-0009] Aubry‐Kientz, M. , Hérault, B. , Ayotte‐Trépanier, C. , Baraloto, C. & Rossi, V. (2013). Toward trait‐based mortality models for tropical forests. PLoS One, 8, e63678. 23675500 10.1371/journal.pone.0063678 PMC3652824

[ele13659-bib-0010] Baraloto, C. , Paine, C.E.T. , Poorter, L. , Beauchene, J. , Bonal, D. , Domenach, A.M. *et al*. (2010). Decoupled leaf and stem economics in rain forest trees. Ecol. Lett., 13, 1338–1347. 20807232 10.1111/j.1461-0248.2010.01517.x

[ele13659-bib-0011] Beeckman, H. (2016). Wood anatomy and trait‐based ecology. IAWA J., 37, 127–151.

[ele13659-bib-0012] Berry, S.L. & Roderick, M.L. (2005). Plant‐water relations and the fibre saturation point. New Phytol., 168, 25–37. 16159318 10.1111/j.1469-8137.2005.01528.x

[ele13659-bib-0013] Brodribb, T.J. , Feild, T.S. & Sack, L. (2010). Viewing leaf structure and evolution from a hydraulic perspective. Funct. Plant Biol., 37, 488–498.

[ele13659-bib-0014] Carmona, C.P. , de Bello, F. , Mason, N.W.H. & Lepš, J. (2016). Traits without borders: integrating functional diversity across scales. Trends Ecol. Evol., 31, 382–394. 26924737 10.1016/j.tree.2016.02.003

[ele13659-bib-0015] Carmona, C.P. , de Bello, F. , Mason, N.W.H. & Lepš, J. (2019). Trait probability density (TPD): measuring functional diversity across scales based on TPD with R. Ecology, 100, 1–8. 10.1002/ecy.2876 31471976

[ele13659-bib-0016] Carmona, C.P. , Rota, C. , Azcárate, F.M. & Peco, B. (2015). More for less: Sampling strategies of plant functional traits across local environmental gradients. Funct. Ecol., 29, 579–588.

[ele13659-bib-0017] Chacón, J.E. & Duong, T. (2018). Multivariate kernel smoothing and its applications, 1st edn. Chapman and Hall/CRC, New York.

[ele13659-bib-0018] Chave, J. , Coomes, D. , Jansen, S. , Lewis, S.L. , Swenson, N.G. & Zanne, A.E. (2009). Towards a worldwide wood economics spectrum. Ecol. Lett., 12, 351–366. 19243406 10.1111/j.1461-0248.2009.01285.x

[ele13659-bib-0019] Condit, R. , Hubbell, S.P. & Foster, R.B. (1996). Changes in tree species abundance in a neotropical forest: Impact of climate change. J. Trop. Ecol., 12, 231–256.

[ele13659-bib-0020] Cunningham, S.A. , Summerhayes, B. & Westoby, M. (1999). Evolutionary divergences in leaf structure and chemistry, comparing rainfall and soil nutrient gradients. Ecol. Monogr., 69, 569–588.

[ele13659-bib-0021] Díaz, S. , Kattge, J. , Cornelissen, J.H.C. , Wright, I.J. , Lavorel, S. , Dray, S. *et al*. (2016). The global spectrum of plant form and function. Nature, 529, 167–171. 26700811 10.1038/nature16489

[ele13659-bib-0022] Dodd, I.C. & Ryan, A.C. (2016). Whole‐Plant Physiological Responses to Water‐Deficit Stress. eLS Plant Science. John Wiley & Sons Ltd, Chichester, pp. 1–9.

[ele13659-bib-0023] Esquivel‐Muelbert, A. , Baker, T.R. , Dexter, K.G. , Lewis, S.L. , Brienen, R.J.W. , Feldpausch, T.R. *et al*. (2019). Compositional response of Amazon forests to climate change. Glob. Chang. Biol., 25, 39–56. 30406962 10.1111/gcb.14413 PMC6334637

[ele13659-bib-0024] Esquivel‐Muelbert, A. , Baker, T.R. , Dexter, K.G. , Lewis, S.L. , ter Steege, H. , Lopez‐Gonzalez, G. *et al*. (2017). Seasonal drought limits tree species across the Neotropics. Ecography (Cop.), 40, 618–629.

[ele13659-bib-0025] Fauset, S. , Baker, T.R. , Lewis, S.L. , Feldpausch, T.R. , Affum‐Baffoe, K. , Foli, E.G. *et al*. (2012). Drought‐induced shifts in the floristic and functional composition of tropical forests in Ghana. Ecol. Lett., 15, 1120–1129. 22812661 10.1111/j.1461-0248.2012.01834.x

[ele13659-bib-0026] Gleason, S.M. , Westoby, M. , Jansen, S. , Choat, B. , Hacke, U.G. , Pratt, R.B. *et al*. (2016). Weak tradeoff between xylem safety and xylem‐specific hydraulic efficiency across the world’s woody plant species. New Phytol., 209, 123–136. 26378984 10.1111/nph.13646

[ele13659-bib-0027] Guevara, H.A. (2001). Propiedades fisicomecánicas de la madera. Universidad Distrital Francisco José de Caldas, Bogotá.

[ele13659-bib-0028] Hacke, U.G. , Sperry, J.S. , Pockman, W.T. , Davis, S.D. & McCulloh, K.A. (2001). Trends in wood density and structure are linked to prevention of xylem implosion by negative pressure. Oecologia, 126, 457–461. 28547229 10.1007/s004420100628

[ele13659-bib-0029] Helmling, S. , Olbrich, A. , Heinz, I. & Koch, G. (2018). Atlas of vessel elements. IAWA J., 39, 250–352.

[ele13659-bib-0030] IAWA , Angyalossy‐Alfonso, V. , Baas, P. , Carlquist, S. , Peres Chimelo, J. & Rauber Coradin, V.T. *et al*. (2007). IAWA list of microscopic features for hardwood identification. IAWA Bull, 4th edn. National Herbarium of the Netherlands, Leiden.

[ele13659-bib-0031] Jacobsen, A.L. , Agenbag, L. , Esler, K.J. , Pratt, R.B. , Ewers, F.W. & Davis, S.D. (2007). Xylem density, biomechanics and anatomical traits correlate with water stress in 17 evergreen shrub species of the Mediterranean‐type climate region of South Africa. J. Ecol., 95, 171–183.

[ele13659-bib-0032] Jacobsen, A.L. , Ewers, F.W. , Pratt, R.B. , Paddock, W.A. & Davis, S.D. (2005). Do xylem fibers affect vessel cavitation resistance? Plant Physiol., 139, 546–556. 16100359 10.1104/pp.104.058404 PMC1203402

[ele13659-bib-0033] Kogan, F. & Guo, W. (2017). Strong 2015–2016 El Niño and implication to global ecosystems from space data. Int. J. Remote Sens., 38, 161–178.

[ele13659-bib-0034] L’Heureux, M.L. , Takahashi, K. , Watkins, A.B. , Barnston, A.G. , Becker, E.J. , Di Liberto, T.E. *et al*. (2017). Observing and predicting the 2015/16 El Niño. Bull. Am. Meteorol. Soc., 98, 1363–1382.

[ele13659-bib-0035] van Laar, A. & Akça, A. (2007). Forest mensuration. In: Managing Forest Ecosystems (eds von Gadow, K. , Pukkala, T. & Tomé, M. )., Springer, Netherlands, p. 283.

[ele13659-bib-0036] Lawlor, D.W. & Tezara, W. (2009). Causes of decreased photosynthetic rate and metabolic capacity in water‐deficient leaf cells: A critical evaluation of mechanisms and integration of processes. Ann. Bot., 103, 561–579. 19155221 10.1093/aob/mcn244 PMC2707350

[ele13659-bib-0037] Li, S. , Lens, F. , Espino, S. , Karimi, Z. , Klepsch, M. , Schenk, H.J. *et al*. (2016). Intervessel pit membrane thickness as a key determinant of embolism resistance in angiosperm xylem. IAWA J., 37, 152–171.

[ele13659-bib-0038] Linares‐Palomino, R. , Oliveira‐Filho, A.T. & Pennington, R.T. (2011). Neotropical seasonally dry forests: diversity, endemism, and biogeography of woody plants. In Seasonally Dry Tropical Forests (eds Dirzo, R. , Young, H.S. , Mooney, H.A. , Ceballos, G. ). Island Press, Washington, DC, pp. 3–21.

[ele13659-bib-0039] Lopezaraiza‐Mikel, M. , Álvarez‐Añorve, M. , Ávila‐Cabadilla, L. , Martén‐Rodríguez, S. , Calvo‐Alvarado, J. , Marcos do Espírito‐Santo, M. et al. (2013). Phenological patterns of tropical dry forests along latitudinal and successional gradients in the Neotropics. In: Tropical Dry Forests in the Americas: Ecology, Conservation, and Management (eds Sanchez‐Azofeifa, A. , Powers, J.S. , Fernandes, G.W. & Quesada, M. ). CRC Press, pp. 101–128.

[ele13659-bib-0040] Madsen, B. & Gamstedt, E.K. (2013). Wood versus plant fibers: Similarities and differences in composite applications. Advances in Materials Science and Engineering, 2013, 1–14.

[ele13659-bib-0041] Markesteijn, L. , Poorter, L. , Bongers, F. , Paz, H. & Sack, L. (2011a). Hydraulics and life history of tropical dry forest tree species: Coordination of species’ drought and shade tolerance. New Phytol., 191, 480–495. 21477008 10.1111/j.1469-8137.2011.03708.x

[ele13659-bib-0042] Markesteijn, L. , Poorter, L. , Paz, H. , Sack, L. & Bongers, F. (2011b). Ecological differentiation in xylem cavitation resistance is associated with stem and leaf structural traits. Plant, Cell Environ., 34, 137–148. 20946587 10.1111/j.1365-3040.2010.02231.x

[ele13659-bib-0043] Maza‐Villalobos, S. , Poorter, L. & Martínez‐Ramos, M. (2013). Effects of ENSO and temporal rainfall variation on the dynamics of successional communities in old‐field succession of a tropical dry forest. PLoS One, 8, e82040. 24349179 10.1371/journal.pone.0082040 PMC3861369

[ele13659-bib-0044] McDowell, N. , Allen, C.D. , Anderson‐Teixeira, K. , Brando, P. , Brienen, R. , Chambers, J. *et al*. (2018). Drivers and mechanisms of tree mortality in moist tropical forests. New Phytol., 219, 851–869. 29451313 10.1111/nph.15027

[ele13659-bib-0045] Méndez‐Alonzo, R. , Paz, H. , Zuluaga, R.C. , Rosell, J.A. & Olson, M.E. (2012). Coordinated evolution of leaf and stem economics in tropical dry forest trees. Ecology, 93, 2397–2406. 23236911 10.1890/11-1213.1

[ele13659-bib-0046] Mendivelso, H.A. , Camarero, J.J. , Royo Obregón, O. , Gutiérrez, E. & Toledo, M. (2013). Differential growth responses to water balance of coexisting deciduous tree species arelinked to wood density in a Bolivian tropical dry forest. PLoS One, 8, e73855. 24116001 10.1371/journal.pone.0073855 PMC3792103

[ele13659-bib-0047] Niinemets, Ü. (2001). Global‐scale climatic controls of leaf dry mass per area, density, and thickness in trees and shrubs. Ecology, 2, 453–469.

[ele13659-bib-0048] Nunes Garcia, B. , Libonati, R. & Nunes, A.M.B. (2018). Extreme drought events over the Amazon Basin: The perspective from the reconstruction of South American Hydroclimate. Water, 10, 1594.

[ele13659-bib-0049] Olson, M.E. & Rosell, J.A. (2013). Vessel diameter‐stem diameter scaling across woody angiosperms and the ecological causes of xylem vessel diameter variation. New Phytol., 197, 1204–1213. 23278439 10.1111/nph.12097

[ele13659-bib-0050] Onoda, Y. , Westoby, M. , Adler, P.B. , Choong, A.M.F. , Clissold, F.J. , Cornelissen, J.H.C. *et al*. (2011). Global patterns of leaf mechanical properties. Ecol. Lett., 14, 301–312. 21265976 10.1111/j.1461-0248.2010.01582.x

[ele13659-bib-0051] Osnas, J.L.D. , Lichstein, J.W. , Reich, P.B. & Pacala, S.W. (2013). Global leaf trait relationships: Mass, area, and the leaf economics spectrum. Science, 340(6133), 741–744.. 23539179 10.1126/science.1231574

[ele13659-bib-0052] Pennington, R.T. , Lavin, M. & Oliveira‐Filho, A. (2009). Woody plant diversity, evolution, and ecology in the tropics: Perspectives from seasonally dry tropical forests. Annu. Rev. Ecol. Evol. Syst., 40, 437–457.

[ele13659-bib-0053] Pennington, R.T. , Lehmann, C.E.R. & Rowland, L.M. (2018). Tropical savannas and dry forests. Curr. Biol., 28, R541–R545. 29738723 10.1016/j.cub.2018.03.014

[ele13659-bib-0054] Pérez‐Harguindeguy, N. , Díaz, S. , Garnier, E. , Lavorel, S. , Poorter, H. , Jaureguiberry, P. *et al*. (2013). New handbook for standardised measurement of plant functional traits worldwide. Aust. J. Bot., 61, 167–234.

[ele13659-bib-0055] Pineda‐García, F. , Paz, H. , Meinzer, F.C. & Angeles, G. (2015). Exploiting water versus tolerating drought: Water‐use strategies of trees in a secondary successional tropical dry forest. Tree Physiol., 36, 208–217. 26687176 10.1093/treephys/tpv124

[ele13659-bib-0056] Pistón, N. , de Bello, F. , Dias, A.T.C. , Götzenberger, L. , Rosado, B.H.P. , de Mattos, E.A. *et al*. (2019). Multidimensional ecological analyses demonstrate how interactions between functional traits shape fitness and life history strategies. J. Ecol., 107, 2317–2328.

[ele13659-bib-0057] Poorter, H. , Niinemets, Ü. , Poorter, L. , Wright, I.J. & Villar, R. (2009). Causes and consequences of variation in leaf mass per area (LMA): A meta‐analysis. New Phytol., 182, 565–588. 19434804 10.1111/j.1469-8137.2009.02830.x

[ele13659-bib-0058] Poorter, L. , McDonald, I. , Alarcón, A. , Fichtler, E. , Licona, J.C. , Peña‐Claros, M. *et al*. (2010). The importance of wood traits and hydraulic conductance for the performance and life history strategies of 42 rainforest tree species. New Phytol., 185, 481–492. 19925555 10.1111/j.1469-8137.2009.03092.x

[ele13659-bib-0059] Poorter, L. , van der Sande, M.T. , Arets, E.J.M.M. , Ascarrunz, N. , Enquist, B. , Finegan, B. *et al*. (2017). Biodiversity and climate determine the functioning of Neotropical forests. Glob. Ecol. Biogeogr., 26, 1423–1434.

[ele13659-bib-0060] Poorter, L. , Wright, S.J. , Paz, H. , Ackerly, D.D. , Condit, R. , Ibarra‐Manríquez, G. *et al*. (2008). Are functional traits good predictors of demographic rates? Evidence from five neotropical forests. Ecology, 89, 1908–1920. 18705377 10.1890/07-0207.1

[ele13659-bib-0061] Powers, J.S. & Tiffin, P. (2010). Plant functional type classifications in tropical dry forests in Costa Rica: Leaf habit versus taxonomic approaches. Funct. Ecol., 24, 927–936.

[ele13659-bib-0062] Powers, J.S. , Vargas, G.G , Brodribb, T.J. , Schwartz, N.B. , Pérez‐Aviles, D. & Smith‐Martin, C.M. *et al*. (2020). A catastrophic tropical drought kills hydraulically vulnerable tree species. Glob. Chang. Biol., 26, 3122–3133. 32053250 10.1111/gcb.15037

[ele13659-bib-0063] Prado‐Junior, J.A. , Schiavini, I. , Vale, V.S. , Arantes, C.S. , van der Sande, M.T. , Lohbeck, M. *et al*. (2016). Conservative species drive biomass productivity in tropical dry forests. J. Ecol., 104, 817–827.

[ele13659-bib-0064] Pratt, R.B. , Jacobsen, A.L. , Ewers, F.W. & Davis, S.D. (2007). Relationships among xylem transport, biomechanics and storage in stems and roots of nine Rhamnaceae species of the California chaparral. New Phytol., 174, 787–798. 17504462 10.1111/j.1469-8137.2007.02061.x

[ele13659-bib-0065] Rosell, J.A. , Olson, M.E. & Anfodillo, T. (2017). Scaling of xylem vessel diameter with plant size: causes, predictions, and outstanding questions. Curr. For. Reports, 3, 46–59.

[ele13659-bib-0066] Salgado‐Negret, B. , Rodríguez, P. , Cabrera, E.N.M. , Ruíz Osorio, C. & Paz, H. (2015). Protocolo para la medicion de rasgos funcionales en plantas. La ecología funcional como aproximación al estudio, manejo y conservación de la biodiversidad: protocolos y aplicaciones. Instituto de Investigación de Recursos Biológicos Alexander von Humboldt, Bogotá, pp. 37–79.

[ele13659-bib-0067] Salleo, S. & Nakdini, A. (2000). Sclerophylly: Evolutionary advantage or mere epiphenomenon? Plant Biosyst., 134, 247–259.

[ele13659-bib-0068] Santiago, L.S. , Goldstein, G. , Meinzer, F.C. , Fisher, J.B. , Machado, K. , Woodruff, D. *et al*. (2004). Leaf photosynthetic traits scale with hydraulic conductivity and wood density in Panamanian forest canopy trees. Oecologia, 140, 543–550. 15232729 10.1007/s00442-004-1624-1

[ele13659-bib-0069] Scholz, A. , Klepsch, M. , Karimi, Z. & Jansen, S. (2013). How to quantify conduits in wood? Front. Plant Sci., 4, 1–11. 23507674 10.3389/fpls.2013.00056 PMC3600434

[ele13659-bib-0070] Silva, J.O. , Espírito‐Santo, M.M. & Morais, H.C. (2015). Leaf traits and herbivory on deciduous and evergreen trees in a tropical dry forest. Basic Appl. Ecol., 16, 210–219.

[ele13659-bib-0071] Slik, J.W.F. (2004). El Niño droughts and their effects on tree species composition and diversity in tropical rain forests. Oecologia, 141, 114–120. 15278429 10.1007/s00442-004-1635-y

[ele13659-bib-0072] Sobrado, M.A. (1997). Embolism vulnerability in drought‐deciduous and evergreen species of a tropical dry forest. Acta Oecologica, 18, 383–391.

[ele13659-bib-0073] Somavilla, N.S. , Kolb, R.M. & Rossatto, D.R. (2014). Leaf anatomical traits corroborate the leaf economic spectrum: a case study with deciduous forest tree species. Rev. Bras. Bot., 37, 69–82.

[ele13659-bib-0074] Sorieul, M. , Dickson, A. , Hill, S.J. & Pearson, H. (2016). Plant fibre: Molecular structure and biomechanical properties, of a complex living material, influencing its deconstruction towards a biobased composite. Materials (Basel), 9, 1–36. 10.3390/ma9080618 PMC5509024 28773739

[ele13659-bib-0075] Stekhoven, D.J. & Bühlmann, P. (2012). MissForest–Non‐parametric missing value imputation for mixed‐type data. Bioinformatics, 28, 112–118. 22039212 10.1093/bioinformatics/btr597

[ele13659-bib-0076] Sterck, F. , Markesteijn, L. , Schieving, F. & Poorter, L. (2011). Functional traits determine trade‐offs and niches in a tropical forest community. Proc. Natl. Acad. Sci. U. S. A., 108, 20627–20632. 22106283 10.1073/pnas.1106950108 PMC3251078

[ele13659-bib-0077] Talbot, J. , Lewis, S.L. , Lopez‐Gonzalez, G. , Brienen, R.J.W. , Monteagudo, A. , Baker, T.R. *et al*. (2014). Methods to estimate aboveground wood productivity from long‐term forest inventory plots. For. Ecol. Manage., 320, 30–38.

[ele13659-bib-0078] Torres, A.M. , Adarve, J.B. , Cárdenas, M. , Vargas, J.A. , Londoño, V. , Rivera, K. *et al*. (2012). Dinámica sucesional de un fragmento de bosque seco tropical del Valle del Cauca. Colombia. Biota Colomb., 13, 66–84.

[ele13659-bib-0079] Traba, J. , Iranzo, E.C. , Carmona, C.P. & Malo, J.E. (2019). Realised niche changes in a native herbivore assemblage associated with the presence of livestock. Oikos, 126, 1400–1409.

[ele13659-bib-0080] Turner, I.M. (1994). Sclerophylly: Primarily protective? Funct. Ecol., 8, 669.

[ele13659-bib-0081] Vicente‐Serrano, S.M. , Zouber, A. , Lasanta, T. & Pueyo, Y. (2012). Dryness is accelerating degradation of vulnerable shrublands in semiarid mediterranean environments. Ecol. Monogr., 82, 407–428.

[ele13659-bib-0082] Wright, I.J. , Reich, P.B. , Westoby, M. , Ackerly, D.D. , Baruch, Z. , Bongers, F. *et al*. (2004). The worldwide leaf economics spectrum. Nature, 428, 821–827. 15103368 10.1038/nature02403

[ele13659-bib-0083] Ziemińska, K. , Westoby, M. & Wright, I.J. (2015). Broad anatomical variation within a narrow wood density range ‐ A study of twig wood across 69 Australian angiosperms. PLoS One, 10, 1–25. 10.1371/journal.pone.0124892 PMC4408027 25906320

