# Supplementary material for: Diverging functional strategies but high sensitivity to an extreme drought in tropical dry forests
Source: Ecol Lett. 2020 Dec 14;24(3):451–63. doi: 10.1111/ele.13659 (PMC9292319; doi:10.1111/ele.13659)
Supplement: Supplementary file 1 — Supplementary Material [file ELE-24-451-s001.docx]

**Diverging functional strategies but high sensitivity to an extreme drought in tropical dry forests**

Roy González-M., Juan M. Posada, Carlos P. Carmona, Fabián Garzón, Viviana Salinas, Álvaro Idárraga-Piedrahita, Camila Pizano, Andrés Avella, René López-Camacho, Natalia Norden, Jhon Nieto, Sandra P. Medina, Gina M. Rodríguez-M., Rebeca Franke-Ante, Alba M. Torres, Rubén Jurado, Hermes Cuadros, Alejandro Castaño-Naranjo, Hernando García and Beatriz Salgado-Negret

**Supporting Information**

**Table S1.** Extended information of the 11 1-ha permanent Tropical Dry Forests (TDF) plots. *Biomass:* Standing biomass (t ha^-1^) estimated as the sum of biomass of all trees for the first census (t_0_). Biomass growth of survivors (BG_S_, t ha^-1^ yr^-1^) was estimated as annual biomass increments resulting from the growth of all trees that survived from t_0_ to the final census (t_fin_). Biomass growth of recruits (BG_R_, t ha^-1^ yr^-1^) was estimated as annual biomass increment obtained from all trees that attained at least 2.5 cm DBH in t_fin_ and were not sampled in t_0_. To avoid biomass overestimation, we consider that each new tree was recruited immediately after t_0_ and assumed that they had an initial DBH of 0 (Talbot *et al.* 2014). Biomass mortality (BM, t ha^-1^ yr^-1^) was estimated as biomass loss from all trees that died between t_0_ and t_fin_; biomass for a DBH of 2.5 cm was subtracted to each dead tree (Talbot *et al.* 2014). Net biomass change (NBC, t ha^-1^ yr^-1^) corresponded to the net annual change in biomass per plot between t_0_ and t_fin_ (Prado-Junior *et al.* 2016; Poorter *et al.* 2017); it was estimated as: NBC = BG_S_ + BG_R_ – BM. The *Standardised Precipitation-Evapotranspiration Index* (SPEI; Vicente-Serrano *et al.* 2012) shows the mean for wet periods (wet), dry periods (dry), and ENSO_2015_. SPEI was calculated based on long-term data from weather stations near the plots (1980 to 2019), and shows the magnitude and strength of drought conditions during the period of analysis, where negative values indicate the SPEI mean for drought periods (red colour) and positive values correspond to wet periods (blue colour). *Climatic conditions*: Total annual rainy days (ARD, no.), aridity index (Aridity, [PET/TAP]), isothermality (Isoth, %), solar radiation (SRad, MJ·m^-1^ x 100), total annual precipitation (TAP, mm), potential evapotranspiration (PET, mm), number of periods with three consecutive dry months (Dry_Periods_, # [three month with <100 mm·month^-1^]), mean annual temperature (MAT, ºC), total precipitation during the three driest months (TP_Driest_ [<100 mm·month^-1^], mm), number of dry months (D_Months_, months with <100 mm·month^-1^), water vapor pressure (WVP, kPa) and wind speed (Wind, m·s^-1^). *Soil conditions*: Acidity (pH), available phosphorus (P, mg·kg^-1^), cation exchange capacity (CEC, cmol^+^·kg^-1^), extractable bases (Ca [Calcium], Mg [Magnesium], K [Potassium], Na [Sodium], cmol^+^·kg^-1^), organic carbon (OC, %) and textural fractions (Sand, Clay, Silt, %). *Land-cover and terrain characteristics*: Surrounding forest area (ha) and topographic roughness (Roughness, %).

| **Study sites** | | |  | **TDF in the Caribbean lowlands’ region** | | |  | **TDF in the Inter**  **Andean region** | | | | | |  |  | **TDF in the dry Savannas** |
| --- | --- | --- | --- | --- | --- | --- | --- | --- | --- | --- | --- | --- | --- | --- | --- | --- |
| **Site name** | | |  | Macuira National Park | Sanctuary of Flora and Fauna Colorados | Tayrona National Park |  | Cardonal Plana Forests | Cardonal Loma Forests | Jabirú Private Natural Reserve | Tambor Private Natural Reserve | Cotove Research Station | El Vinculo Regional Park | Taminango Research Station |  | Tuparro National Park |
| **Latitude** (ºN) | | |  | 12.20 | 9.94 | 11.31 |  | 5.08 | 5.09 | 5.06 | 5.17 | 6.53 | 3.84 | 1.67 |  | 5.25 |
| **Longitude** (ºW) | | |  | -71.35 | -75.11 | -74.13 |  | -74.80 | -74.77 | -74.83 | -74.81 | -75.83 | -76.29 | -77.31 |  | -67.86 |
| **Altitude (masl)** | | |  | 113 | 301 | 15 |  | 260 | 322 | 302 | 385 | 509 | 1025 | 591 |  | 95 |
| **Number of species** (#ha) | | |  | 34 | 66 | 53 |  | 49 | 47 | 35 | 77 | 28 | 45 | 9 |  | 76 |
| **Deciduous/Evergreen** | | |  | 24/10 | 30/36 | 34/19 |  | 21/28 | 22/25 | 15/20 | 27/50 | 15/13 | 19/26 | 5/4 |  | 22/54 |
| **Standing biomass** | | |  | 80.9 | 103.8 | 110.7 |  | 87.6 | 132.9 | 106.1 | 116.1 | 76.9 | 62.0 | 19.9 |  | 73.8 |
| **BG_S_** | | |  | 1.45 | 2.68 | 1.85 |  | 1.65 | 2.63 | 3.59 | 2.58 | 2.44 | 2.22 | 1.18 |  | 2.25 |
| **BG_R_** | | |  | 0.002 | 0.007 | 0.048 |  | 0.212 | 0.027 | 0.360 | 0.166 | 0.178 | 0.087 | 0.020 |  | 0.007 |
| **BM** | | |  | 0.63 | 0.63 | 0.40 |  | 0.87 | 1.01 | 1.45 | 0.66 | 0.41 | 0.59 | 0.25 |  | 0.30 |
| **BNC** | | |  | 0.82 | 2.06 | 1.49 |  | 0.99 | 1.64 | 2.50 | 2.09 | 2.21 | 1.73 | 0.95 |  | 1.96 |
| **Weather stations**  (Δt=1980-2019) | | |  | 2 | | |  | 4 | | | | | | |  | 1 |
| **SPEI wet** | | |  | 0.82 | | |  | 0.81 | | | | | | |  | 0.74 |
| **SPEI dry** | | |  | -0.77 | | |  | -0.80 | | | | | | |  | -0.80 |
| **SPEI ENSO**_2015_  (Δt=May/2015-Jan/2016) | | |  | -1.42 | | |  | -1.21 | | | | | | |  | -1.87 |
| **Climate** | | |  |  |  |  |  |  |  |  |  |  |  |  |  |  |
|  | Ard | |  | 33 | 96 | 95 |  | 116 | 113 | 116 | 126 | 146 | 144 | 138 |  | 152 |
|  | Aridity | |  | 3.42 | 1.01 | 2.03 |  | 1.29 | 1.19 | 1.31 | 0.88 | 1.43 | 0.97 | 1.94 |  | 0.77 |
|  | Dry_Periods_ | |  | 1 | 1 | 1 |  | 2 | 2 | 2 | 2 | 1 | 2 | 2 |  | 1 |
|  | D_Months_ | |  | 10 | 5 | 9 |  | 5 | 5 | 5 | 4 | 6 | 5 | 9 |  | 4 |
|  | Isoth | |  | 75.43 | 90.25 | 81.35 |  | 85.97 | 85.91 | 86.03 | 85.83 | 87.29 | 93.30 | 91.78 |  | 78.07 |
|  | MAT | |  | 27.11 | 26.1 | 27.38 |  | 27.86 | 27.44 | 27.86 | 26.81 | 26.92 | 23.38 | 25.25 |  | 28.25 |
|  | SRad | |  | 185.69 | 192.08 | 196.79 |  | 173.36 | 172.78 | 173.28 | 172.87 | 178.49 | 169.95 | 159.45 |  | 164.10 |
|  | TAP | |  | 517.0 | 1528.4 | 899.4 |  | 1505.9 | 1541.2 | 1528.2 | 1912.5 | 1193.8 | 1192.4 | 721.4 |  | 2697.2 |
|  | PET | |  | 1768.6 | 1546.0 | 1827.7 |  | 1946.8 | 1835.6 | 2009.1 | 1689.1 | 1712.8 | 1161.3 | 1400.8 |  | 2067.0 |
|  | TP_Driest_ | |  | 32.1 | 139.3 | 33.4 |  | 227.8 | 222.5 | 236.8 | 272.7 | 112.7 | 168.5 | 52.4 |  | 177.1 |
|  | WVP | |  | 2.67 | 2.75 | 2.87 |  | 2.63 | 2.53 | 2.65 | 2.50 | 2.55 | 2.14 | 2.36 |  | 2.80 |
|  | Wind | |  | 4.84 | 2.35 | 4.45 |  | 0.93 | 0.92 | 0.93 | 0.90 | 0.83 | 0.87 | 0.96 |  | 1.37 |
| **Soils** | | |  |  |  |  |  |  |  |  |  |  |  |  |  |  |
|  | pH | |  | 6.12 | 7.37 | 7.38 |  | 6.79 | 6.87 | 6.46 | 6.98 | 6.54 | 6.22 | 7.23 |  | 4.39 |
|  | P | |  | 18.14 | 12.17 | 222.59 |  | 143.24 | 19.36 | 17.08 | 11.33 | 20.08 | 4.70 | 27.89 |  | 3.32 |
|  | CEC | |  | 10.01 | 30.89 | 16.43 |  | 15.61 | 20.08 | 17.01 | 14.73 | 25.71 | 26.68 | 33.10 |  | 6.69 |
|  | Extractable Bases | |  |  |  |  |  |  |  |  |  |  |  |  |  |  |
|  |  | Ca |  | 5.29 | 34.83 | 16.05 |  | 13.81 | 22.89 | 13.13 | 10.29 | 22.13 | 21.33 | 29.14 |  | 0.06 |
|  |  | Mg |  | 2.19 | 4.64 | 2.69 |  | 3.24 | 4.11 | 3.90 | 2.30 | 9.95 | 17.74 | 7.91 |  | 0.06 |
|  |  | K |  | 0.55 | 0.65 | 0.82 |  | 0.73 | 0.36 | 0.70 | 0.61 | 0.38 | 0.87 | 1.08 |  | 0.21 |
|  |  | Na |  | 0.22 | 0.09 | 0.07 |  | 0.03 | 0.10 | 0.08 | 0.04 | 0.15 | 0.14 | 0.16 |  | 0.16 |
|  | OC | |  | 1.00 | 3.22 | 3.58 |  | 2.54 | 2.41 | 2.80 | 2.36 | 2.26 | 3.64 | 2.61 |  | 1.79 |
|  | Textural fractions | |  |  |  |  |  |  |  |  |  |  |  |  |  |  |
|  |  | Sand |  | 57.41 | 34.21 | 62.58 |  | 61.86 | 56.45 | 48.86 | 72.25 | 37.42 | 60.69 | 34.47 |  | 64.27 |
|  |  | Clay |  | 21.54 | 31.58 | 19.25 |  | 24.96 | 24.54 | 21.16 | 16.33 | 35.34 | 24.36 | 29.66 |  | 16.39 |
|  |  | Silt |  | 21.07 | 34.21 | 18.18 |  | 13.18 | 19.03 | 29.98 | 11.42 | 27.24 | 14.95 | 35.9 |  | 19.34 |
| **Forest area** | | |  | 407.6 | 395.0 | 411.8 |  | 153.9 | 303.0 | 145.4 | 329.6 | 55.1 | 23.4 | 56.8 |  | 176.3 |
| **Roughness** | | |  | 10.4 | 14.7 | 19.1 |  | 7.3 | 12.7 | 9.0 | 22.5 | 9.7 | 8.0 | 20.8 |  | 7.4 |

**Figure S1.** Pairwise correlations between imputed individual-trait values (IMP) and not-imputed individual-trait values. *Functional traits:* Fibre wall thickness (FWT, μm), hydraulically weighted diameter (d_h_, μm), leaf area (LA, mm^2^), leaf dry matter content (LDMC, mg g^-1^), leaf thickness (L_th_, mm), maximum vessel area (VA_max_, μm^2^), pit area (PA, μm^2^), pit diameter aperture (DA_pit_, μm), specific leaf area (SLA, mm^2^ mg^-1^), vessel area (VA, μm^2^), vessel density (VD, vessels mm^-2^), wood density (WD, g cm^3^), anhydrous wood density (WD_0_, g cm^3^), water content at maximum capacity (WC_max_, kg kg^-1^), and xylem potential hydraulic conductivity (K_s_, kg m^-1^ s^-1^ MPa^-1^).

**Figure S2.** Conceptual framework showing the steps to calculate Trait Probability Densities (TPD) in this study. In the example, we used four populations (P; in total we had 524 populations) belonging to three species (sp; in total we had 338 species) sampled in four plots (p; in total we had 11 plots). Here, a population refers to all sampled individuals (i) of the same species within a plot, for both traits and biomass. *Step 1*. To describe each individual's functional characterization in a 2-dimensional trait space, we performed a PCA analysis that reduces the matrix of sampled traits (in total 15 functional traits) to the principal axes of variation. The PCA analysis provides the uncorrelated and compressed first components as the new trait values (_PCA_trait_1_ and _PCA_trait_2_). In this study, the first PCA axis (36.75% of explained variance) reflected the hydraulic safety-efficiency trade-off. Negative values in PC1, high safety, describe species having a high density of narrow vessels with high fibre wall thickness, whereas positive values, hydraulic efficiency, characterized species having large vessels and pits with high xylem potential hydraulic conductivity. The second PCA axis (24.57% of explained variance) reflected differences of investment in tissues, where negative values were related to large leaves with high SLA, and high content of water at maximal capacity (‘cheap’ tissues), while positive values corresponded to high LDMC and high wood density (‘costly’ tissues). *Step 2*. TPD was calculated following the procedures suggested by Carmona *et al.* (2016) based on the bivariate gaussian kernel density functions. In the example, we first calculated the TPD of each population (TPD_P_) as the sum of all individual trait density probabilities (TPD_i_). We then summed all bivariate density functions for populations (TPD_P_) to obtain the trait probability density for all species in TDF (TPD_C_). *Step 3*. To calculated the TPD_C_ rescaled by biomass (TPD_Cs_) each TPD_P_ was multiplying by the relative biomass of each population. As the relative biomass sum to 1 across all TDF populations, the sum of all the rescaled TPDP functions’ integrals was 1. We made this procedure for all biomass dimensions used in this study (e.g., standing biomass–SB, biomass growth of survivors, biomass growth of recruits, and net biomass change). *Step 4*. At last, we calculated the functional trait space occupied by the TPD_C_ and TPD_Cs_ at three probability thresholds (20%, 50%, and 99%) using the *Functional Richness* (F_Ric_) index suggested by Carmona *et al.* (2016, 2019).

**Table S2.** Functional trait space scores and biomass values for 524 populations belonging to 338 species in TDF. *Functional trait space*: The first PCA axis (PC1, 36.75% of explained variance) reflected the hydraulic safety-efficiency trade-off; negative values in PC1 corresponded to high safety for species having a high density of narrow vessels with high fibre wall thickness, whereas positive values corresponded to high hydraulic efficiency, characterized by species having large vessels and pits, with high xylem potential hydraulic conductivity. The second PCA axis (PC2, 24.57% of explained variance) reflected differences in tissue investment, where negative values were related to large leaves with high SLA, and high content of water at maximal capacity (‘cheap’ tissues), while positive values refereed to high LDMC and high wood density (‘costly’ tissues). *Biomass*: Standing biomass (kg ha^-1^) refers to the sum of biomass for all trees of each species in each plot for the first census (t_0_). Biomass growth of survivors (BG_S_, kg ha^-1^ yr^-1^) refers to the annual biomass increment produced by the growth of all trees of each species that survived from t_0_ to the final census (t_fin_) in a plot. Biomass growth of recruits (BG_R_, kg ha^-1^ yr^-1^) refers to the annual biomass increment obtained from all trees of each species that attained at least 2.5 cm DBH in t_fin_ and that were not sampled in t_0_ in a plot. Biomass mortality (BM, kg ha^-1^ yr^-1^) refers to the biomass loss obtained from all trees of each species between t_0_ and t_fin_. Net biomass change (NBC, kg ha^-1^ yr^-1^) refers to the net annual change in biomass during the time interval between t_0_ and t_fin_ (Prado-Junior *et al.* 2016; Poorter *et al.* 2017). Values were rounded to two decimals. Voucher code (v) and individual tagged number (tag) are provided when a species was not fully identified.

| **Study sites** | | **Family** | **Species** | **PC1** | **PC2** | **Standing Biomass** | **BG_S_** | **BG_R_** | **BM** | **NBC** |
| --- | --- | --- | --- | --- | --- | --- | --- | --- | --- | --- |
| **Caribbean lowlands’ region** | | | | | |  |  |  |  |  |
|  | **Macuira National Park** | | | | |  |  |  |  |  |
|  |  | Anacardiaceae | *Astronium graveolens* | 0.72 | 0.86 | 18409.31 | 343.19 | 0.00 | 124.88 | 218.31 |
|  |  | Bignoniaceae | *Handroanthus billbergii* | 0.77 | 0.89 | 27936.60 | 632.26 | 0.00 | 24.00 | 608.26 |
|  |  | Bignoniaceae | *Handroanthus chrysanthus* | 0.20 | -0.17 | 90.30 | 3.63 | 0.00 | 0.00 | 3.63 |
|  |  | Boraginaceae | *Cordia alba* | 0.85 | -0.85 | 83.04 | 0.57 | 0.00 | 0.00 | 0.57 |
|  |  | Boraginaceae | *Cordia macuirensis* | -0.74 | 0.24 | 1.11 | 0.00 | 0.00 | 0.00 | 0.00 |
|  |  | Burseraceae | *Bursera graveolens* | 0.84 | -1.26 | 3455.96 | 45.83 | 0.00 | 19.35 | 26.48 |
|  |  | Burseraceae | *Bursera simaruba* | 0.77 | -1.27 | 4258.10 | 103.30 | 0.00 | 14.87 | 88.43 |
|  |  | Capparaceae | *Capparidastrum pachaca* | -1.18 | 1.09 | 312.30 | 0.00 | 0.00 | 29.44 | -29.44 |
|  |  | Capparaceae | *Capparis* sp2 [v. HC-6659, tag. 129] | -0.91 | 0.24 | 44.91 | 0.31 | 0.00 | 4.99 | -4.68 |
|  |  | Capparaceae | *Cynophalla linearis* | -0.80 | 1.26 | 2715.36 | 30.16 | 0.00 | 30.49 | -0.34 |
|  |  | Capparaceae | *Cynophalla verrucosa* | -0.93 | 0.66 | 17.67 | 0.31 | 0.00 | 3.24 | -2.92 |
|  |  | Euphorbiaceae | *Croton punctatus* | -1.21 | -0.33 | 3.27 | 0.14 | 0.00 | 0.00 | 0.14 |
|  |  | Fabaceae | *Erythrina velutina* | 1.46 | -1.42 | 4361.96 | 18.78 | 0.00 | 29.57 | -10.79 |
|  |  | Fabaceae | *Lonchocarpus pictus* | 1.21 | 1.00 | 4629.47 | 45.76 | 1.40 | 149.13 | -101.97 |
|  |  | Fabaceae | *Lonchocarpus violaceus* | 2.14 | 1.42 | 177.17 | 1.57 | 0.00 | 0.00 | 1.57 |
|  |  | Fabaceae | *Machaerium arboreum* | 0.95 | 0.66 | 4397.31 | 24.01 | 0.00 | 112.22 | -88.21 |
|  |  | Fabaceae | *Myrospermum frutescens* | 0.89 | 1.05 | 214.38 | 5.19 | 0.00 | 0.00 | 5.19 |
|  |  | Fabaceae | *Myrospermum* sp [tag. 578] | 0.51 | 1.29 | 60.49 | 0.41 | 0.00 | 0.00 | 0.41 |
|  |  | Fabaceae | *Pithecellobium dulce* | 0.77 | 0.44 | 14.03 | 0.57 | 0.00 | 0.00 | 0.57 |
|  |  | Fabaceae | *Prosopis juliflora* | 0.59 | 0.81 | 628.57 | 49.89 | 0.00 | 8.54 | 41.35 |
|  |  | Hernandiaceae | *Gyrocarpus americanus* | 0.82 | -2.07 | 878.74 | 11.22 | 0.00 | 13.42 | -2.21 |
|  |  | Malpighiaceae | *Bunchosia odorata* | -0.90 | 0.27 | 31.31 | 1.82 | 0.12 | 5.48 | -3.54 |
|  |  | Malvaceae | *Pachira quinata* | 1.54 | -0.60 | 2490.44 | 35.96 | 0.00 | 0.00 | 35.96 |
|  |  | Nyctaginaceae | *Neea* sp [tag. 707] | -0.23 | 0.04 | 113.45 | 1.38 | 0.00 | 0.00 | 1.38 |
|  |  | Polygonaceae | *Coccoloba caracasana* | -0.84 | 0.59 | 811.09 | 24.73 | 0.00 | 0.00 | 24.73 |
|  |  | Polygonaceae | *Coccoloba* sp [tag. 218] | -0.24 | 0.55 | 1715.88 | 21.97 | 0.00 | 18.11 | 3.86 |
|  |  | Rubiaceae | *Chiococca* sp [v. HC-6652, tag. 461] | -1.92 | -0.04 | 120.51 | 1.75 | 0.00 | 7.83 | -6.07 |
|  |  | Rubiaceae | *Coutarea* sp [v. HC-6641, tag. 35] | -1.81 | 0.37 | 62.18 | 1.89 | 0.00 | 2.70 | -0.81 |
|  |  | Rubiaceae | *Randia aculeata* | -1.72 | 0.54 | 230.77 | 4.58 | 0.00 | 19.10 | -14.51 |
|  |  | Rutaceae | *Zanthoxylum schreberi* | -1.57 | 0.65 | 280.67 | 3.10 | 0.00 | 7.19 | -4.09 |
|  |  | Salicaceae | *Casearia* sp6 [v. HC-6635, tag. 262] | -1.64 | -0.19 | 133.73 | 0.79 | 0.00 | 8.48 | -7.69 |
|  |  | Sapindaceae | *Melicoccus bijugatus* | 0.26 | 0.34 | 717.19 | 2.06 | 0.00 | 0.00 | 2.06 |
|  |  | Sapindaceae | *Melicoccus oliviformis* | 1.05 | 1.07 | 958.62 | 13.98 | 0.00 | 0.00 | 13.98 |
|  |  | Sapotaceae | *Pradosia colombiana* | -0.13 | 1.23 | 560.42 | 22.72 | 0.00 | 0.00 | 22.72 |
|  | **Sanctuary of Flora and Fauna Colorados** | | | | |  |  |  |  |  |
|  |  | Achariaceae | *Mayna grandifolia* | -0.60 | 0.22 | 3.45 | 0.53 | 0.00 | 0.00 | 0.53 |
|  |  | Achatocarpaceae | *Achatocarpus nigricans* | -0.68 | -1.27 | 477.08 | 15.90 | 0.00 | 0.85 | 15.06 |
|  |  | Anacardiaceae | *Astronium graveolens* | 0.64 | 0.18 | 25.21 | 0.93 | 0.00 | 0.00 | 0.93 |
|  |  | Anacardiaceae | *Spondias radlkoferi* | -0.02 | -1.27 | 2035.18 | 38.86 | 0.00 | 9.52 | 29.34 |
|  |  | Annonaceae | *Oxandra* sp [v. HC-6491, tag. 13] | -0.86 | 0.75 | 2041.64 | 39.03 | 1.06 | 34.36 | 5.73 |
|  |  | Apocynaceae | *Aspidosperma polyneuron* | -1.21 | 0.37 | 2565.56 | 92.31 | 0.00 | 1.77 | 90.54 |
|  |  | Apocynaceae | *Aspidosperma* sp [tag. 2991] | 0.94 | 0.35 | 0.00 | 0.00 | 0.00 | 0.00 | 0.00 |
|  |  | Apocynaceae | *Tabernaemontana cymosa* | -1.19 | -1.18 | 20.21 | 0.07 | 0.00 | 0.00 | 0.07 |
|  |  | Apocynaceae | *Tabernaemontana* sp1 [v. HC-6532, tag. 202] | -0.28 | -3.49 | 365.03 | 7.03 | 0.02 | 4.84 | 2.21 |
|  |  | Asteraceae | *Chromolaena perglabra* | -1.34 | -0.11 | 305.73 | 0.94 | 0.00 | 12.05 | -11.11 |
|  |  | Burseraceae | *Bursera simaruba* | -0.20 | -3.06 | 2274.64 | 15.26 | 0.00 | 63.36 | -48.09 |
|  |  | Capparaceae | *Cynophalla verrucosa* | -1.01 | 0.48 | 2158.41 | 40.76 | 0.03 | 14.89 | 25.89 |
|  |  | Capparaceae | *Quadrella indica* | -0.33 | -0.81 | 136.08 | 0.33 | 0.00 | 0.00 | 0.33 |
|  |  | Capparaceae | *Quadrella odoratissima* | -0.30 | 0.44 | 1002.69 | 9.76 | 0.00 | 5.15 | 4.61 |
|  |  | Euphorbiaceae | *Hura crepitans* | 1.33 | -0.29 | 4.45 | 0.07 | 0.00 | 0.00 | 0.07 |
|  |  | Fabaceae | *Albizia* sp2 [v. HC-6494, tag. 81] | 0.78 | 0.29 | 336.48 | 20.87 | 0.00 | 0.00 | 20.87 |
|  |  | Fabaceae | *Coursetia ferruginea* | 1.16 | 0.16 | 13037.52 | 32.53 | 0.00 | 6.43 | 26.10 |
|  |  | Fabaceae | *Inga vera* | 0.80 | -0.20 | 19.81 | 5.07 | 0.00 | 0.00 | 5.07 |
|  |  | Fabaceae | *Peltogyne purpurea* | 0.29 | 1.15 | 13.26 | 0.00 | 0.00 | 0.00 | 0.00 |
|  |  | Fabaceae | *Peltogyne* sp [v. HC-6497, tag. 31] | 0.05 | 0.73 | 2291.65 | 22.04 | 0.00 | 69.46 | -47.42 |
|  |  | Fabaceae | *Platymiscium pinnatum* | 0.78 | 0.48 | 1096.60 | 77.17 | 0.00 | 0.00 | 77.17 |
|  |  | Fabaceae | *Pterocarpus rohrii* | 0.19 | 0.13 | 196.01 | 1.59 | 0.23 | 0.60 | 1.22 |
|  |  | Fabaceae | *Senegalia* sp [v. HC-6506, tag. 44] | 0.80 | -0.62 | 1753.04 | 32.52 | 0.00 | 57.33 | -24.81 |
|  |  | Fabaceae | *Swartzia simplex* | -0.06 | 0.65 | 2.24 | 0.00 | 0.04 | 0.00 | 0.04 |
|  |  | Fabaceae | *Zygia* sp [tag. 836] | 0.67 | 1.49 | 95.74 | 0.00 | 0.00 | 0.00 | 0.00 |
|  |  | Lamiaceae | *Aegiphila* sp [v. HC-6531, tag. 221] | 1.34 | -0.57 | 14.18 | 1.33 | 0.00 | 0.00 | 1.33 |
|  |  | Lamiaceae | *Vitex* sp [tag. 307] | -0.46 | 0.01 | 2.52 | 0.00 | 0.00 | 0.00 | 0.00 |
|  |  | Lecythidaceae | *Gustavia superba* | -0.98 | -0.65 | 154.51 | 5.70 | 0.07 | 0.73 | 5.04 |
|  |  | Lecythidaceae | *Lecythis minor* | 0.05 | 0.12 | 0.00 | 0.00 | 0.00 | 0.00 | 0.00 |
|  |  | Malpighiaceae | *Malpighia glabra* | -0.97 | -0.25 | 26.00 | 2.11 | 0.00 | 0.00 | 2.11 |
|  |  | Malvaceae | *Cavanillesia platanifolia* | 1.23 | -1.58 | 5827.36 | 1.58 | 0.00 | 0.00 | 1.58 |
|  |  | Malvaceae | *Ceiba pentandra* | 1.89 | -0.77 | 5318.54 | 0.00 | 0.00 | 0.00 | 0.00 |
|  |  | Malvaceae | *Guazuma ulmifolia* | 0.78 | -1.07 | 246.46 | 10.04 | 0.00 | 0.00 | 10.04 |
|  |  | Malvaceae | *Pachira quinata* | -0.34 | -3.85 | 66.58 | 2.03 | 0.00 | 1.25 | 0.78 |
|  |  | Meliaceae | *Trichilia acuminata* | -1.01 | 1.09 | 6437.26 | 116.53 | 2.00 | 122.73 | -4.20 |
|  |  | Meliaceae | *Trichilia elegans* | -0.86 | -0.19 | 186.96 | 3.71 | 0.00 | 0.00 | 3.71 |
|  |  | Moraceae | *Brosimum alicastrum* | 0.71 | 0.61 | 2335.12 | 680.01 | 1.10 | 7.69 | 673.42 |
|  |  | Moraceae | *Brosimum* sp [v. HC-6558, tag. 1613] | 0.48 | 0.54 | 7426.03 | 121.18 | 0.00 | 25.31 | 95.87 |
|  |  | Moraceae | *Sorocea sprucei* | 0.54 | 0.53 | 1333.92 | 61.54 | 0.00 | 2.17 | 59.37 |
|  |  | Myrtaceae | *Eugenia procera* | -1.14 | 0.98 | 599.88 | 10.68 | 0.12 | 5.88 | 4.92 |
|  |  | Nyctaginaceae | *Guapira* sp [v. HC-6578, tag. 974] | -0.01 | -0.73 | 142.22 | 1.49 | 0.00 | 0.00 | 1.49 |
|  |  | Phyllanthaceae | *Margaritaria nobilis* | -0.01 | -0.07 | 17.73 | 0.00 | 0.00 | 0.00 | 0.00 |
|  |  | Polygonaceae | *Coccoloba padiformis* | -0.05 | 0.59 | 3.02 | 0.18 | 0.08 | 0.00 | 0.27 |
|  |  | Polygonaceae | *Coccoloba* sp1 [v. HC-6507, tag. 52] | 0.33 | 0.86 | 3111.72 | 167.95 | 0.00 | 1.51 | 166.44 |
|  |  | Primulaceae | *Ardisia foetida* | -0.26 | -0.49 | 48.89 | 0.50 | 0.09 | 1.31 | -0.71 |
|  |  | Rubiaceae | *Alibertia* sp [v. HC-6536, tag. 236] | -0.55 | 0.40 | 15.30 | 0.00 | 0.00 | 0.00 | 0.00 |
|  |  | Rubiaceae | *Chiococca* sp [v. HC-6595, tag. 270] | -1.50 | 0.29 | 40.93 | 0.06 | 0.00 | 0.00 | 0.06 |
|  |  | Rubiaceae | *Coutarea hexandra* | -0.97 | -0.92 | 15.42 | 2.17 | 0.00 | 0.00 | 2.17 |
|  |  | Rubiaceae | *Morf* sp17 [tag. 1001] | -0.63 | -2.49 | 35.07 | 0.00 | 0.00 | 0.00 | 0.00 |
|  |  | Rubiaceae | *Pittoniotis* sp [tag. 1190] | 0.17 | -0.38 | 131.74 | 0.00 | 0.00 | 0.00 | 0.00 |
|  |  | Rubiaceae | *Pittoniotis trichantha* | -0.64 | 0.09 | 2279.77 | 18.46 | 0.00 | 25.37 | -6.90 |
|  |  | Rubiaceae | *Rudgea* sp [v. HC-6599, tag. 1065] | -1.01 | 0.16 | 55.10 | 2.42 | 0.13 | 0.00 | 2.55 |
|  |  | Rutaceae | *Amyris pinnata* | -1.11 | 0.07 | 1291.98 | 50.45 | 0.04 | 0.00 | 50.49 |
|  |  | Rutaceae | *Esenbeckia pentaphylla* | -0.89 | -0.04 | 5109.60 | 267.80 | 0.00 | 2.96 | 264.83 |
|  |  | Rutaceae | *Galipea* sp [tag. 1236] | -0.63 | 0.51 | 144.94 | 9.17 | 0.03 | 0.00 | 9.20 |
|  |  | Rutaceae | *Zanthoxylum* sp4 [v. HC-6494, tag. 17] | -1.10 | 0.75 | 1078.15 | 32.41 | 0.00 | 13.09 | 19.32 |
|  |  | Salicaceae | *Casearia sylvestris* | -1.15 | -0.39 | 8.72 | 0.65 | 0.00 | 0.00 | 0.65 |
|  |  | Sapindaceae | *Allophylus* sp [v. HC-6619, tag. 159] | -0.76 | -0.14 | 0.00 | 0.00 | 0.00 | 0.00 | 0.00 |
|  |  | Sapindaceae | *Melicoccus bijugatus* | 0.71 | 1.50 | 29.25 | 1.29 | 0.00 | 0.00 | 1.29 |
|  |  | Sapindaceae | *Melicoccus oliviformis* | 1.05 | 1.06 | 74.68 | 4.13 | 0.00 | 0.00 | 4.13 |
|  |  | Sapotaceae | *Manilkara* sp [tag. 318] | 0.05 | 1.27 | 211.63 | 10.99 | 0.00 | 0.00 | 10.99 |
|  |  | Sapotaceae | *Pouteria* sp1 [v. HC-6508, tag. 739] | -0.41 | 0.87 | 5949.59 | 95.35 | 0.28 | 39.00 | 56.62 |
|  |  | Sapotaceae | *Pouteria* sp2 [tag. 955] | 0.13 | 0.19 | 0.00 | 0.00 | 0.00 | 0.00 | 0.00 |
|  |  | Sapotaceae | *Pouteria* sp3 [tag. 163] | -0.40 | 0.93 | 79.45 | 2.24 | 0.00 | 0.00 | 2.24 |
|  |  | Sapotaceae | *Pouteria* sp7 [v. HC-6480, tag. 124] | -0.53 | 0.91 | 351.82 | 9.93 | 0.04 | 14.39 | -4.41 |
|  |  | Sapotaceae | *Pradosia colombiana* | 0.06 | 0.64 | 3054.36 | 53.25 | 0.00 | 0.00 | 53.25 |
|  |  | Stemonuraceae | *Discophora* sp [v. HC-6557, tag. 581] | -0.85 | 0.42 | 118.59 | 3.66 | 0.00 | 0.00 | 3.66 |
|  |  | Ulmaceae | *Ampelocera macphersonii* | 0.12 | 0.61 | 16791.18 | 435.47 | 2.07 | 82.40 | 355.14 |
|  |  | Zygophyllaceae | *Bulnesia arborea* | 0.03 | 1.09 | 1394.12 | 38.86 | 0.00 | 0.00 | 38.86 |
|  | **Tayrona National Park** | | | | |  |  |  |  |  |
|  |  | Anacardiaceae | *Astronium graveolens* | 0.71 | 0.72 | 15716.00 | 388.46 | 0.07 | 0.00 | 388.53 |
|  |  | Anacardiaceae | *Spondias mombin* | -0.28 | -1.68 | 5446.50 | 58.83 | 0.00 | 6.18 | 52.64 |
|  |  | Asteraceae | *Chromolaena perglabra* | -1.39 | -1.06 | 26.77 | 0.15 | 0.00 | 1.46 | -1.31 |
|  |  | Boraginaceae | *Cordia alba* | 0.20 | -1.18 | 335.60 | 6.29 | 11.15 | 6.16 | 11.28 |
|  |  | Boraginaceae | *Cordia* sp [v. HC-6723, tag. 355] | 0.15 | -0.55 | 4.37 | 0.32 | 0.11 | 0.00 | 0.43 |
|  |  | Burseraceae | *Bursera simaruba* | 0.65 | -1.93 | 2812.19 | 82.75 | 0.00 | 14.64 | 68.11 |
|  |  | Capparaceae | *Capparidastrum pachaca* | -0.28 | 0.90 | 3562.54 | 59.14 | 2.98 | 5.38 | 56.73 |
|  |  | Capparaceae | *Capparidastrum tenuisiliquum* | -1.21 | -0.18 | 1072.35 | 12.74 | 7.69 | 10.22 | 10.20 |
|  |  | Capparaceae | *Crateva tapia* | -0.01 | -0.79 | 130.66 | 1.32 | 0.00 | 0.00 | 1.32 |
|  |  | Capparaceae | *Cynophalla flexuosa* | -0.19 | 0.82 | 273.64 | 3.65 | 0.00 | 3.70 | -0.05 |
|  |  | Capparaceae | *Cynophalla verrucosa* | -0.98 | 0.59 | 2666.03 | 39.82 | 4.67 | 12.16 | 32.33 |
|  |  | Capparaceae | *Quadrella indica* | -0.54 | 0.12 | 278.74 | 5.71 | 0.05 | 0.00 | 5.76 |
|  |  | Capparaceae | *Quadrella odoratissima* | -0.13 | 0.78 | 14742.47 | 81.11 | 1.45 | 15.39 | 67.17 |
|  |  | Erythroxylaceae | *Erythroxylum hondense* | -1.61 | 0.49 | 54.78 | 0.66 | 0.00 | 0.00 | 0.66 |
|  |  | Euphorbiaceae | *Croton niveus* | -0.74 | 0.11 | 970.89 | 6.56 | 1.76 | 37.91 | -29.59 |
|  |  | Euphorbiaceae | *Hura crepitans* | 1.58 | -1.37 | 2791.77 | 13.54 | 0.00 | 0.00 | 13.54 |
|  |  | Euphorbiaceae | *Manihot carthaginensis* | 2.78 | -0.43 | 45.26 | 2.72 | 0.00 | 0.00 | 2.72 |
|  |  | Fabaceae | *Albizia niopoides* | 1.06 | 0.62 | 809.64 | 22.77 | 5.29 | 2.80 | 25.26 |
|  |  | Fabaceae | *Caesalpinia punctata* | 1.65 | 1.74 | 4546.64 | 39.54 | 0.00 | 0.00 | 39.54 |
|  |  | Fabaceae | *Coursetia ferruginea* | 1.14 | -0.21 | 203.30 | 11.40 | 0.71 | 2.24 | 9.87 |
|  |  | Fabaceae | *Machaerium capote* | 1.40 | 0.48 | 938.48 | 170.91 | 0.00 | 0.64 | 170.27 |
|  |  | Fabaceae | *Machaerium* sp1 [v. HC-6729, tag. 144] | 1.32 | 0.41 | 961.96 | 11.58 | 0.00 | 2.64 | 8.94 |
|  |  | Fabaceae | *Muellera broadwayi* | 1.61 | 0.42 | 2510.94 | 26.68 | 0.00 | 0.00 | 26.68 |
|  |  | Fabaceae | *Pithecellobium roseum* | 0.41 | 1.40 | 148.99 | 0.70 | 0.00 | 2.84 | -2.14 |
|  |  | Fabaceae | *Platymiscium pinnatum* | 1.28 | 1.11 | 1925.33 | 16.85 | 0.00 | 0.45 | 16.40 |
|  |  | Fabaceae | *Prosopis juliflora* | 0.58 | 0.30 | 1391.18 | 29.63 | 0.00 | 0.00 | 29.63 |
|  |  | Fabaceae | *Pterocarpus rohrii* | 0.77 | 0.24 | 19481.61 | 323.73 | 0.60 | 145.43 | 178.90 |
|  |  | Fabaceae | *Senegalia tamarindifolia* | 0.44 | 0.18 | 1261.20 | 30.78 | 2.28 | 34.57 | -1.50 |
|  |  | Fabaceae | *Senna atomaria* | 1.10 | 0.56 | 167.96 | 5.72 | 0.00 | 0.00 | 5.72 |
|  |  | Fabaceae | *Senna* sp [tag. 272] | 1.18 | -0.10 | 116.60 | 3.69 | 0.00 | 0.00 | 3.69 |
|  |  | Fabaceae | *Senna* sp1 [v. HC-6714, tag. 1179] | 0.43 | 0.39 | 247.13 | 3.63 | 0.00 | 3.48 | 0.15 |
|  |  | Fabaceae | *Vachellia farnesiana* | 0.76 | 0.99 | 588.53 | 18.60 | 0.46 | 11.23 | 7.84 |
|  |  | Hernandiaceae | *Gyrocarpus americanus* | -0.25 | -1.33 | 894.85 | 8.84 | 0.00 | 0.00 | 8.84 |
|  |  | Indet | Morf sp14 [tag. 746] | -0.58 | 0.43 | 11.76 | 0.97 | 0.00 | 0.00 | 0.97 |
|  |  | Lecythidaceae | *Eschweilera* sp [v. HC-6681, tag. 109] | -1.90 | 0.34 | 81.97 | 0.58 | 0.09 | 0.00 | 0.67 |
|  |  | Malvaceae | *Guazuma ulmifolia* | -0.28 | -0.84 | 273.76 | 4.78 | 0.00 | 2.70 | 2.08 |
|  |  | Malvaceae | *Pseudobombax septenatum* | 1.03 | -2.03 | 738.37 | 77.31 | 0.05 | 0.00 | 77.37 |
|  |  | Moraceae | *Brosimum alicastrum* | 0.20 | 0.16 | 7.89 | 0.08 | 0.00 | 0.61 | -0.53 |
|  |  | Myrtaceae | *Eugenia* sp1 [v. HC-6699, tag. 731] | -0.88 | 1.39 | 3.21 | 0.06 | 0.00 | 0.00 | 0.06 |
|  |  | Nyctaginaceae | *Guapira* sp [tag. 335] | -0.51 | -0.75 | 67.03 | 1.22 | 2.77 | 0.54 | 3.44 |
|  |  | Nyctaginaceae | *Guapira uberrima* | -0.67 | -0.55 | 1418.54 | 10.24 | 0.27 | 36.49 | -25.98 |
|  |  | Polygonaceae | *Coccoloba obtusifolia* | -0.24 | -0.03 | 528.40 | 13.33 | 0.61 | 8.45 | 5.49 |
|  |  | Polygonaceae | *Triplaris americana* | -0.03 | -0.60 | 201.87 | 1.25 | 0.00 | 0.00 | 1.25 |
|  |  | Primulaceae | *Bonellia frutescens* | -1.18 | -0.02 | 197.15 | 1.02 | 0.00 | 0.54 | 0.48 |
|  |  | Rubiaceae | *Calycophyllum candidissimum* | -0.32 | 0.00 | 227.01 | 3.62 | 0.46 | 2.00 | 2.08 |
|  |  | Rubiaceae | *Psychotria* sp [v. HC-6751, tag. 1375] | -1.26 | -1.23 | 2.57 | 0.15 | 0.00 | 0.00 | 0.15 |
|  |  | Rubiaceae | *Randia aculeata* | -1.20 | 0.43 | 18.45 | 0.12 | 0.00 | 0.00 | 0.12 |
|  |  | Rubiaceae | *Simira cordifolia* | -1.46 | -0.50 | 8.78 | 0.10 | 0.00 | 0.00 | 0.10 |
|  |  | Salicaceae | *Casearia praecox* | -1.65 | -0.37 | 141.08 | 3.21 | 0.00 | 0.80 | 2.41 |
|  |  | Sapindaceae | *Melicoccus bijugatus* | 1.23 | 0.84 | 7216.29 | 104.82 | 4.17 | 0.00 | 108.99 |
|  |  | Sapindaceae | *Melicoccus oliviformis* | 0.66 | 0.96 | 4161.97 | 93.63 | 0.22 | 0.00 | 93.85 |
|  |  | Sapindaceae | *Sapindus saponaria* | 0.77 | 0.77 | 109.50 | 10.52 | 0.00 | 0.00 | 10.52 |
|  |  | Sapotaceae | *Pradosia colombiana* | -0.21 | 0.63 | 8115.70 | 31.90 | 0.00 | 30.86 | 1.04 |
| **Inter Andean region** | | | | | |  |  |  |  |  |
|  | **Cardonal Loma Forests** | | | | |  |  |  |  |  |
|  |  | Achariaceae | *Mayna odorata* | -1.17 | 0.65 | 2284.78 | 50.21 | 2.84 | 70.86 | -17.81 |
|  |  | Anacardiaceae | *Astronium graveolens* | 0.98 | 0.65 | 21467.34 | 409.48 | 0.00 | 55.33 | 354.15 |
|  |  | Apocynaceae | *Aspidosperma polyneuron* | -0.61 | 0.69 | 19754.58 | 413.49 | 0.15 | 48.91 | 364.73 |
|  |  | Bignoniaceae | *Tabebuia rosea* | 1.13 | 1.15 | 3876.59 | 113.01 | 0.00 | 0.00 | 113.01 |
|  |  | Boraginaceae | *Cordia gerascanthus* | 0.44 | 0.54 | 1314.39 | 17.22 | 4.81 | 22.97 | -0.94 |
|  |  | Burseraceae | *Bursera simaruba* | 1.37 | -0.73 | 4669.39 | 64.98 | 0.00 | 47.15 | 17.83 |
|  |  | Capparaceae | *Cynophalla polyantha* | 0.34 | 1.26 | 496.55 | 8.59 | 0.00 | 0.00 | 8.59 |
|  |  | Capparaceae | *Quadrella odoratissima* | 0.22 | 1.17 | 658.24 | 11.19 | 0.00 | 0.00 | 11.19 |
|  |  | Ebenaceae | *Diospyros* sp2 [v. RG-1910, tag. 2731] | 0.27 | 0.70 | 3.62 | 0.15 | 0.00 | 0.00 | 0.15 |
|  |  | Euphorbiaceae | *Croton schiedeanus* | -0.36 | 0.06 | 159.04 | 3.04 | 0.04 | 2.18 | 0.90 |
|  |  | Euphorbiaceae | *Croton* sp [v. RG-1874, tag. 245] | 0.03 | 1.11 | 19.60 | 0.00 | 0.00 | 0.00 | 0.00 |
|  |  | Fabaceae | *Bauhinia petiolata* | -0.57 | 1.25 | 1626.90 | 48.15 | 6.01 | 46.87 | 7.29 |
|  |  | Fabaceae | *Calliandra magdalenae* | -0.01 | 1.39 | 880.81 | 11.15 | 0.00 | 46.40 | -35.25 |
|  |  | Fabaceae | *Machaerium capote* | 0.81 | 0.54 | 9203.82 | 137.01 | 1.61 | 63.81 | 74.82 |
|  |  | Fabaceae | *Machaerium* sp1 [v. RG-1891, tag. 368] | 1.19 | 1.08 | 711.25 | 9.26 | 0.00 | 7.06 | 2.21 |
|  |  | Fabaceae | *Machaerium* sp6 [v. RG-1850, tag. 2917] | 1.02 | 0.80 | 3414.98 | 28.98 | 0.39 | 93.39 | -64.02 |
|  |  | Fabaceae | *Platymiscium pinnatum* | 1.34 | 0.96 | 3485.97 | 59.63 | 0.00 | 27.60 | 32.03 |
|  |  | Fabaceae | *Pterocarpus rohrii* | 0.92 | 0.69 | 10523.74 | 185.76 | 0.00 | 91.40 | 94.35 |
|  |  | Fabaceae | *Senegalia* sp1 [v. RG-1895, tag. 996] | 1.75 | 0.21 | 902.60 | 15.07 | 1.28 | 36.85 | -20.50 |
|  |  | Fabaceae | *Swartzia trianae* | -0.26 | 0.80 | 3054.22 | 49.11 | 0.02 | 21.91 | 27.22 |
|  |  | Malpighiaceae | *Bunchosia* sp [v. RG-1894, tag. 981] | -1.57 | 0.58 | 4.93 | 0.11 | 0.00 | 0.00 | 0.11 |
|  |  | Malpighiaceae | *Malpighia glabra* | -0.99 | 0.34 | 142.82 | 1.71 | 0.05 | 12.60 | -10.84 |
|  |  | Malvaceae | *Pseudobombax septenatum* | 1.89 | -1.11 | 4245.40 | 14.96 | 0.00 | 0.00 | 14.96 |
|  |  | Meliaceae | *Trichilia carinata* | -0.95 | 0.47 | 1076.77 | 31.80 | 0.59 | 0.00 | 32.39 |
|  |  | Meliaceae | *Trichilia elegans* | -0.49 | 0.59 | 3937.68 | 79.62 | 0.77 | 37.35 | 43.04 |
|  |  | Meliaceae | *Trichilia oligofoliolata* | -1.38 | 0.85 | 12045.54 | 442.70 | 4.53 | 25.55 | 421.69 |
|  |  | Meliaceae | *Trichilia pallida* | -0.97 | 0.22 | 4405.16 | 74.92 | 0.02 | 25.26 | 49.69 |
|  |  | Myrtaceae | *Eugenia procera* | -1.51 | 1.03 | 1545.02 | 46.44 | 2.40 | 8.16 | 40.68 |
|  |  | Myrtaceae | Morf sp1 [v. RG-1881, tag. 376] | -1.22 | 1.23 | 1.75 | 0.00 | 0.00 | 0.00 | 0.00 |
|  |  | Nyctaginaceae | *Neea* sp1 [v. RG-1871, tag. 161] | -0.12 | 0.24 | 208.16 | 1.81 | 0.00 | 11.87 | -10.06 |
|  |  | Polygonaceae | *Coccoloba* sp1 [v. RG-1843, tag. 2103] | -0.09 | 1.64 | 3112.52 | 62.82 | 0.84 | 2.86 | 60.80 |
|  |  | Polygonaceae | *Ruprechtia* sp1 [v. RG-1914, tag. 3445] | -0.31 | -0.53 | 1.84 | 0.20 | 0.00 | 0.00 | 0.20 |
|  |  | Polygonaceae | *Triplaris melaenodendron* | 0.73 | 0.20 | 1378.28 | 11.83 | 0.37 | 34.78 | -22.58 |
|  |  | Rhamnaceae | *Ziziphus strychnifolia* | 0.42 | 0.88 | 1189.79 | 11.16 | 0.00 | 13.78 | -2.62 |
|  |  | Rubiaceae | *Guettarda comata* | -0.92 | 0.05 | 393.97 | 3.37 | 0.00 | 1.98 | 1.40 |
|  |  | Rubiaceae | *Randia aculeata* | -1.48 | -0.12 | 3.79 | 0.00 | 0.00 | 0.82 | -0.82 |
|  |  | Rubiaceae | *Randia armata* | -1.03 | 0.41 | 630.48 | 10.75 | 0.00 | 34.21 | -23.46 |
|  |  | Rutaceae | *Amyris pinnata* | -0.91 | 0.64 | 1032.39 | 23.31 | 0.00 | 21.35 | 1.96 |
|  |  | Rutaceae | *Esenbeckia alata* | -1.55 | 0.92 | 3.62 | 0.05 | 0.00 | 0.00 | 0.05 |
|  |  | Rutaceae | *Zanthoxylum* sp2 [v. RG-1848, tag. 2890] | -0.40 | -0.09 | 781.82 | 30.46 | 0.00 | 6.73 | 23.73 |
|  |  | Salicaceae | *Casearia corymbosa* | -1.39 | 0.68 | 784.87 | 6.10 | 0.00 | 6.54 | -0.44 |
|  |  | Salicaceae | *Casearia praecox* | -1.79 | 0.28 | 1182.97 | 11.29 | 0.03 | 0.29 | 11.03 |
|  |  | Salicaceae | *Casearia* sp1 [v. RG-1897, tag. 1086] | -1.81 | 0.35 | 39.69 | 0.18 | 0.00 | 0.00 | 0.18 |
|  |  | Salicaceae | *Casearia sylvestris* | -1.60 | 0.45 | 4714.76 | 96.70 | 0.00 | 74.45 | 22.25 |
|  |  | Sapotaceae | *Pouteria* sp7 [v. RG-1865, tag. 111] | -0.27 | 0.73 | 1254.55 | 21.70 | 0.11 | 12.97 | 8.84 |
|  |  | Ulmaceae | *Ampelocera* sp1 [v. RG-1870, tag. 166] | 0.16 | 1.23 | 141.07 | 8.85 | 0.00 | 0.00 | 8.85 |
|  |  | Violaceae | *Rinorea* sp1 [tag. 788] | -0.91 | 1.34 | 114.25 | 2.17 | 0.00 | 0.00 | 2.17 |
|  | **Cardonal Plana Forests** | | | | |  |  |  |  |  |
|  |  | Achariaceae | *Mayna odorata* | -1.25 | 0.54 | 2754.22 | 46.62 | 0.00 | 126.23 | -79.61 |
|  |  | Achatocarpaceae | *Achatocarpus nigricans* | -0.70 | -0.35 | 2796.34 | 66.47 | 15.62 | 36.78 | 45.31 |
|  |  | Anacardiaceae | *Astronium graveolens* | 1.10 | 0.48 | 7433.11 | 99.65 | 0.22 | 43.32 | 56.54 |
|  |  | Anacardiaceae | *Spondias mombin* | 2.40 | -0.45 | 111.97 | 0.61 | 0.00 | 0.00 | 0.61 |
|  |  | Annonaceae | *Oxandra espintana* | -0.45 | 0.37 | 4184.72 | 66.75 | 45.21 | 47.11 | 64.84 |
|  |  | Apocynaceae | *Aspidosperma polyneuron* | -0.99 | 0.57 | 117.44 | 1.37 | 0.00 | 0.00 | 1.37 |
|  |  | Bignoniaceae | *Handroanthus chrysanthus* | 0.73 | -0.06 | 357.53 | 0.68 | 0.00 | 0.00 | 0.68 |
|  |  | Bignoniaceae | *Tabebuia rosea* | 0.39 | 0.06 | 397.18 | 15.92 | 0.00 | 0.00 | 15.92 |
|  |  | Boraginaceae | *Cordia gerascanthus* | 0.18 | -0.06 | 872.97 | 13.91 | 0.00 | 28.09 | -14.18 |
|  |  | Boraginaceae | *Cordia* sp [v. RG-1938, tag. 248] | -0.64 | -0.64 | 60.73 | 0.74 | 0.00 | 5.42 | -4.69 |
|  |  | Capparaceae | *Capparidastrum frondosum* | 0.04 | 0.52 | 0.00 | 0.00 | 0.00 | 0.00 | 0.00 |
|  |  | Capparaceae | *Morisonia americana* | 0.37 | 0.69 | 43.91 | 0.00 | 0.00 | 0.00 | 0.00 |
|  |  | Ebenaceae | *Diospyros* sp2 [v. RG-1957, tag. 472] | 0.32 | -0.16 | 130.60 | 0.89 | 0.00 | 0.00 | 0.89 |
|  |  | Fabaceae | *Bauhinia petiolata* | -0.94 | 1.06 | 1041.69 | 18.28 | 0.00 | 20.00 | -1.71 |
|  |  | Fabaceae | *Calliandra magdalenae* | 0.19 | 1.14 | 2016.74 | 55.83 | 2.24 | 24.74 | 33.34 |
|  |  | Fabaceae | *Machaerium capote* | 0.99 | 0.43 | 7619.72 | 179.43 | 11.26 | 17.83 | 172.86 |
|  |  | Fabaceae | *Machaerium* sp1 [tag. 1525] | 1.11 | 0.98 | 1.19 | 0.07 | 0.00 | 0.00 | 0.07 |
|  |  | Fabaceae | *Pterocarpus rohrii* | 1.61 | 0.20 | 1046.32 | 9.66 | 0.02 | 0.00 | 9.68 |
|  |  | Fabaceae | *Senegalia* sp1 [tag. 458] | 0.48 | -0.66 | 8141.87 | 171.32 | 0.65 | 122.91 | 49.07 |
|  |  | Fabaceae | *Swartzia trianae* | 0.54 | 0.15 | 7312.02 | 170.46 | 0.00 | 5.93 | 164.52 |
|  |  | Lamiaceae | *Aegiphila* sp1 [tag. 832] | 0.63 | -1.01 | 458.57 | 8.03 | 0.00 | 0.00 | 8.03 |
|  |  | Lauraceae | *Ocotea veraguensis* | 0.64 | 0.57 | 5356.22 | 27.54 | 0.16 | 37.93 | -10.23 |
|  |  | Lecythidaceae | *Gustavia* sp [v. RG-1917, tag. 7] | -0.89 | 0.67 | 803.96 | 12.26 | 37.94 | 26.13 | 24.08 |
|  |  | Malpighiaceae | *Malpighia glabra* | -1.46 | 0.19 | 57.34 | 1.68 | 0.07 | 1.97 | -0.21 |
|  |  | Malvaceae | *Guazuma ulmifolia* | 0.68 | -1.41 | 47.85 | 2.86 | 0.12 | 0.00 | 2.98 |
|  |  | Meliaceae | *Trichilia carinata* | -1.05 | 0.52 | 4816.82 | 87.28 | 7.54 | 54.14 | 40.69 |
|  |  | Meliaceae | *Trichilia elegans* | -0.89 | 0.41 | 515.18 | 11.54 | 0.03 | 6.68 | 4.90 |
|  |  | Meliaceae | *Trichilia oligofoliolata* | -1.39 | 0.98 | 79.21 | 3.49 | 0.00 | 0.00 | 3.49 |
|  |  | Meliaceae | *Trichilia pallida* | -0.80 | -0.47 | 3059.99 | 32.61 | 0.05 | 35.52 | -2.85 |
|  |  | Moraceae | *Brosimum alicastrum* | 0.94 | -0.03 | 30.71 | 0.00 | 0.00 | 0.00 | 0.00 |
|  |  | Myrtaceae | *Eugenia procera* | -1.20 | 1.08 | 743.38 | 16.29 | 0.12 | 13.44 | 2.97 |
|  |  | Myrtaceae | *Eugenia* sp5 [v. RG-1928, tag. 1901] | -0.84 | 1.36 | 259.65 | 0.78 | 10.27 | 21.40 | -10.35 |
|  |  | Nyctaginaceae | *Neea* sp1 [tag. 1414] | 0.25 | 0.46 | 10.70 | 0.08 | 0.00 | 1.23 | -1.15 |
|  |  | Polygonaceae | *Coccoloba acuminata* | 0.06 | 0.51 | 19.50 | 0.11 | 0.00 | 0.00 | 0.11 |
|  |  | Polygonaceae | *Coccoloba* sp1 [tag. 30] | -0.94 | 1.21 | 3298.38 | 45.58 | 11.87 | 21.13 | 36.32 |
|  |  | Polygonaceae | *Ruprechtia* sp1 [v. RG-1941, tag. 47] | -0.01 | -0.70 | 1422.93 | 33.43 | 0.09 | 0.00 | 33.52 |
|  |  | Polygonaceae | *Triplaris melaenodendron* | 0.64 | 0.12 | 4160.65 | 66.97 | 11.88 | 53.36 | 25.49 |
|  |  | Rhamnaceae | *Ziziphus strychnifolia* | 1.33 | 0.64 | 2835.82 | 40.88 | 0.92 | 4.42 | 37.38 |
|  |  | Rubiaceae | *Randia armata* | -0.94 | 0.47 | 798.70 | 37.34 | 49.60 | 25.19 | 61.75 |
|  |  | Rubiaceae | *Simira cordifolia* | -0.95 | 0.37 | 3744.38 | 69.36 | 0.85 | 57.17 | 13.04 |
|  |  | Rutaceae | *Esenbeckia alata* | -0.99 | 1.01 | 1455.53 | 9.88 | 0.00 | 0.00 | 9.88 |
|  |  | Rutaceae | *Zanthoxylum rhoifolium* | -0.41 | -0.54 | 462.96 | 22.03 | 0.00 | 9.98 | 12.05 |
|  |  | Rutaceae | *Zanthoxylum rigidum* | -0.77 | -0.13 | 1.13 | 0.29 | 0.00 | 0.00 | 0.29 |
|  |  | Salicaceae | *Casearia corymbosa* | -1.57 | -0.16 | 33.32 | 1.60 | 0.00 | 0.00 | 1.60 |
|  |  | Salicaceae | *Casearia praecox* | -1.81 | 0.17 | 133.88 | 2.18 | 0.00 | 0.00 | 2.18 |
|  |  | Salicaceae | *Casearia sylvestris* | -1.03 | 0.50 | 1592.36 | 22.26 | 0.00 | 14.56 | 7.70 |
|  |  | Sapotaceae | *Pouteria* sp7 [tag. 10] | -0.24 | 1.02 | 2867.13 | 51.62 | 0.17 | 4.45 | 47.33 |
|  |  | Sapotaceae | *Pouteria* sp8 [v. RG-1925, tag. 1629] | 0.29 | 0.95 | 70.93 | 72.65 | 0.00 | 0.00 | 72.65 |
|  |  | Ulmaceae | *Ampelocera* sp1 [v. RG-1918, tag. 6] | 0.70 | 1.49 | 2061.80 | 53.54 | 5.29 | 5.53 | 53.31 |
|  | **Cotove Research Station** | | | | |  |  |  |  |  |
|  |  | Achatocarpaceae | *Achatocarpus nigricans* | -1.26 | -1.52 | 267.39 | 9.68 | 0.00 | 1.72 | 7.95 |
|  |  | Anacardiaceae | *Astronium graveolens* | 1.04 | -0.34 | 3618.92 | 30.89 | 0.08 | 51.48 | -20.52 |
|  |  | Apocynaceae | *Tabernaemontana grandiflora* | -1.68 | -2.14 | 18.04 | 0.00 | 0.00 | 3.78 | -3.78 |
|  |  | Araliaceae | *Aralia excelsa* | -0.36 | -2.53 | 0.00 | 0.00 | 0.00 | 0.00 | 0.00 |
|  |  | Burseraceae | *Bursera simaruba* | 0.64 | -2.18 | 3768.94 | 26.65 | 0.11 | 28.94 | -2.18 |
|  |  | Capparaceae | *Quadrella indica* | -0.23 | -0.21 | 218.43 | 10.02 | 0.19 | 5.43 | 4.78 |
|  |  | Erythroxylaceae | *Erythroxylum hondense* | -1.12 | 0.28 | 1.20 | 0.10 | 0.00 | 0.00 | 0.10 |
|  |  | Fabaceae | *Enterolobium cyclocarpum* | 0.93 | -1.48 | 6953.70 | 4.02 | 0.00 | 60.81 | -56.79 |
|  |  | Fabaceae | *Leucaena leucocephala* | 1.04 | -0.68 | 2162.75 | 7.03 | 0.00 | 71.77 | -64.75 |
|  |  | Fabaceae | *Platymiscium pinnatum* | 0.28 | -0.25 | 139.58 | 1.49 | 0.00 | 0.00 | 1.49 |
|  |  | Fabaceae | *Pseudosamanea guachapele* | 0.95 | -0.67 | 3250.49 | 16.61 | 0.00 | 60.55 | -43.94 |
|  |  | Indet. | Morf sp6 [tag. 461] | -1.05 | -0.18 | 3.33 | 0.05 | 0.00 | 0.00 | 0.05 |
|  |  | Malpighiaceae | *Bunchosia armeniaca* | 0.10 | 0.58 | 388.21 | 2.67 | 0.00 | 7.73 | -5.06 |
|  |  | Malpighiaceae | *Malpighia glabra* | -0.60 | -0.22 | 1208.35 | 15.56 | 2.39 | 3.00 | 14.94 |
|  |  | Malvaceae | *Ceiba pentandra* | 2.27 | -1.15 | 1077.99 | 52.46 | 0.00 | 0.85 | 51.61 |
|  |  | Moraceae | *Brosimum alicastrum* | 0.71 | 0.90 | 70.42 | 0.58 | 0.00 | 0.00 | 0.58 |
|  |  | Moraceae | *Castilla elastica* | 0.49 | -2.49 | 0.95 | 0.00 | 0.00 | 0.46 | -0.46 |
|  |  | Myrtaceae | *Eugenia venezuelensis* | -0.08 | 1.02 | 2.92 | 0.10 | 0.00 | 0.00 | 0.10 |
|  |  | Phyllanthaceae | *Phyllanthus botryanthus* | -0.75 | -0.50 | 568.43 | 13.02 | 2.79 | 17.77 | -1.96 |
|  |  | Rubiaceae | *Chomelia spinosa* | -1.18 | -0.81 | 93.40 | 0.40 | 0.00 | 2.07 | -1.67 |
|  |  | Rutaceae | *Amyris pinnata* | -0.20 | 0.49 | 372.87 | 4.06 | 0.00 | 8.64 | -4.58 |
|  |  | Rutaceae | *Zanthoxylum fagara* | -0.04 | -0.20 | 1169.75 | 23.07 | 0.40 | 37.14 | -13.68 |
|  |  | Rutaceae | *Zanthoxylum lenticulare* | 0.20 | -0.65 | 1119.22 | 31.23 | 170.26 | 6.38 | 195.11 |
|  |  | Rutaceae | *Zanthoxylum schreberi* | -0.46 | -0.73 | 1466.26 | 21.07 | 1.31 | 9.11 | 13.27 |
|  |  | Salicaceae | *Casearia corymbosa* | -2.15 | 0.28 | 39.57 | 0.30 | 0.00 | 2.80 | -2.51 |
|  |  | Salicaceae | *Casearia praecox* | -0.32 | -0.06 | 104.51 | 0.71 | 0.05 | 8.82 | -8.06 |
|  |  | Sapindaceae | *Melicoccus bijugatus* | 0.80 | 0.45 | 48647.07 | 2165.66 | 0.61 | 16.57 | 2149.70 |
|  |  | Sapindaceae | *Sapindus saponaria* | 1.80 | -0.17 | 120.06 | 2.96 | 0.00 | 0.00 | 2.96 |
|  | **Jabirú Private Natural Reserve** | | | | |  |  |  |  |  |
|  |  | Achariaceae | *Mayna odorata* | -1.21 | -0.58 | 78.59 | 9.41 | 0.00 | 0.00 | 9.41 |
|  |  | Anacardiaceae | *Astronium graveolens* | 0.24 | -0.56 | 5465.49 | 72.52 | 0.00 | 12.98 | 59.54 |
|  |  | Annonaceae | *Oxandra espintana* | -1.42 | -0.21 | 8387.17 | 427.89 | 2.32 | 195.58 | 234.63 |
|  |  | Apocynaceae | *Aspidosperma polyneuron* | -1.34 | -0.15 | 183.57 | 7.77 | 0.00 | 0.00 | 7.77 |
|  |  | Bignoniaceae | *Handroanthus chrysanthus* | 0.09 | -0.21 | 1263.06 | 29.48 | 0.00 | 43.38 | -13.90 |
|  |  | Boraginaceae | *Cordia gerascanthus* | -0.25 | -0.67 | 206.10 | 9.33 | 0.00 | 0.00 | 9.33 |
|  |  | Burseraceae | *Bursera simaruba* | 0.22 | -2.61 | 635.96 | 11.06 | 0.00 | 0.00 | 11.06 |
|  |  | Capparaceae | *Cynophalla flexuosa* | -0.25 | -0.44 | 45.38 | 1.05 | 0.00 | 0.00 | 1.05 |
|  |  | Capparaceae | *Cynophalla polyantha* | -0.64 | -0.51 | 389.28 | 6.57 | 0.00 | 0.00 | 6.57 |
|  |  | Ebenaceae | *Diospyros* sp2 [v. JAC-2202, tag. 1830] | 0.11 | 0.65 | 399.30 | 4.89 | 0.00 | 0.00 | 4.89 |
|  |  | Fabaceae | *Machaerium capote* | -0.09 | -0.70 | 8017.24 | 114.57 | 0.00 | 37.15 | 77.42 |
|  |  | Fabaceae | *Piptadenia* sp [v. JAC-2187, tag. 1196] | 0.25 | -0.62 | 1166.38 | 0.14 | 0.00 | 90.05 | -89.91 |
|  |  | Fabaceae | *Platymiscium pinnatum* | 1.06 | 0.20 | 219.90 | 9.82 | 0.00 | 0.00 | 9.82 |
|  |  | Fabaceae | *Swartzia trianae* | -0.25 | 0.28 | 255.91 | 11.52 | 0.00 | 0.00 | 11.52 |
|  |  | Lauraceae | *Ocotea veraguensis* | -0.07 | -0.21 | 863.26 | 9.31 | 0.00 | 0.00 | 9.31 |
|  |  | Malvaceae | *Pseudobombax septenatum* | -0.01 | -2.51 | 1285.20 | 15.41 | 0.00 | 0.00 | 15.41 |
|  |  | Meliaceae | *Trichilia carinata* | -1.06 | 0.07 | 3734.54 | 153.10 | 0.00 | 216.59 | -63.49 |
|  |  | Meliaceae | *Trichilia oligofoliolata* | -1.54 | 0.34 | 53642.81 | 2172.34 | 357.75 | 265.73 | 2264.36 |
|  |  | Meliaceae | *Trichilia pallida* | -1.38 | -0.73 | 3215.02 | 82.06 | 0.00 | 46.27 | 35.79 |
|  |  | Myrtaceae | *Eugenia procera* | -1.40 | 0.45 | 2261.94 | 119.08 | 0.28 | 121.55 | -2.19 |
|  |  | Nyctaginaceae | *Guapira* sp [v. JAC-2204, tag. 2498] | -0.15 | -1.84 | 78.79 | 1.91 | 0.00 | 7.04 | -5.13 |
|  |  | Polygonaceae | *Coccoloba* sp1 [tag. 1275] | -0.38 | 0.68 | 4.65 | 0.38 | 0.00 | 0.00 | 0.38 |
|  |  | Polygonaceae | *Coccoloba* sp2 [v. JAC-2171, tag. 618] | -0.71 | 0.51 | 5832.28 | 115.60 | 0.00 | 174.90 | -59.30 |
|  |  | Polygonaceae | *Ruprechtia* sp1 [v. JAC-2189, tag. 1093] | -0.41 | -1.37 | 547.98 | 6.98 | 0.00 | 0.00 | 6.98 |
|  |  | Polygonaceae | *Triplaris melaenodendron* | 0.64 | -0.82 | 1775.49 | 51.12 | 0.00 | 38.85 | 12.26 |
|  |  | Rubiaceae | *Randia armata* | -1.41 | -0.23 | 832.59 | 23.45 | 0.00 | 24.14 | -0.69 |
|  |  | Rubiaceae | *Randia dioica* | -0.33 | 0.06 | 7.39 | 1.10 | 0.00 | 0.00 | 1.10 |
|  |  | Rutaceae | *Amyris pinnata* | -1.70 | -0.67 | 0.00 | 0.00 | 0.00 | 0.00 | 0.00 |
|  |  | Rutaceae | *Zanthoxylum rhoifolium* | -0.27 | -2.33 | 416.47 | 11.06 | 0.00 | 35.83 | -24.77 |
|  |  | Rutaceae | *Zanthoxylum rigidum* | -0.34 | -0.44 | 25.32 | 0.87 | 0.00 | 0.00 | 0.87 |
|  |  | Rutaceae | *Zanthoxylum schreberi* | -0.48 | -0.44 | 146.79 | 0.00 | 0.00 | 2.74 | -2.74 |
|  |  | Salicaceae | *Casearia praecox* | -1.59 | -0.60 | 1023.44 | 12.53 | 0.00 | 121.45 | -108.92 |
|  |  | Salicaceae | *Casearia* sp1 [tag. 157] | -1.70 | -0.58 | 538.81 | 19.35 | 0.00 | 0.00 | 19.35 |
|  |  | Sapotaceae | *Pouteria* sp7 [v. JAC-2152, tag. 120] | -0.97 | -0.14 | 1750.20 | 42.08 | 0.00 | 6.23 | 35.86 |
|  |  | Ulmaceae | *Ampelocera* sp1 [v. JAC-2154, tag. 79] | -0.23 | 0.20 | 1057.49 | 32.49 | 0.00 | 6.29 | 26.21 |
|  |  | Violaceae | *Leonia* sp1 [v. JAC-2207, tag. 2494] | -0.51 | -2.31 | 374.12 | 1.72 | 0.00 | 0.00 | 1.72 |
|  | **Tambor Private Natural Reserve** | | | | |  |  |  |  |  |
|  |  | Achariaceae | *Mayna odorata* | -0.68 | -0.47 | 87.78 | 5.61 | 0.00 | 0.38 | 5.22 |
|  |  | Anacardiaceae | *Anacardium excelsum* | 1.54 | -1.48 | 66199.73 | 790.50 | 0.04 | 0.19 | 790.35 |
|  |  | Anacardiaceae | *Astronium graveolens* | 1.15 | -0.44 | 3939.02 | 45.08 | 0.34 | 0.00 | 45.42 |
|  |  | Anacardiaceae | *Spondias mombin* | 2.06 | -1.74 | 3243.11 | 49.98 | 0.00 | 0.00 | 49.98 |
|  |  | Annonaceae | *Malmea* sp [v. JAC-3209, tag. 167] | 0.54 | 0.57 | 201.93 | 0.29 | 0.00 | 0.00 | 0.29 |
|  |  | Annonaceae | *Oxandra espintana* | 0.20 | 0.63 | 87.49 | 2.60 | 0.00 | 0.00 | 2.60 |
|  |  | Annonaceae | *Pseudomalmea* sp [v. RLC-15595, tag. 7] | 0.14 | -0.56 | 1012.64 | 23.11 | 0.20 | 17.00 | 6.32 |
|  |  | Annonaceae | *Rollinia mucosa* | 1.41 | -1.20 | 134.12 | 13.26 | 1.08 | 2.56 | 11.78 |
|  |  | Apocynaceae | *Aspidosperma* sp1 [v. RLC-15602, tag. 30] | 0.05 | 0.03 | 0.00 | 0.00 | 0.00 | 0.00 | 0.00 |
|  |  | Apocynaceae | *Tabernaemontana grandiflora* | -1.33 | -1.63 | 798.04 | 18.76 | 2.09 | 17.88 | 2.97 |
|  |  | Apocynaceae | *Tabernaemontana markgrafiana* | -1.58 | -1.10 | 2.09 | 0.11 | 0.00 | 0.00 | 0.11 |
|  |  | Bignoniaceae | *Jacaranda caucana* | 0.58 | -1.31 | 1599.37 | 0.06 | 0.97 | 0.00 | 1.03 |
|  |  | Bignoniaceae | *Tabebuia rosea* | 0.86 | -0.35 | 3805.51 | 28.44 | 0.38 | 58.03 | -29.21 |
|  |  | Boraginaceae | *Cordia alliodora* | 0.47 | -0.05 | 16.16 | 11.77 | 0.00 | 0.00 | 11.77 |
|  |  | Boraginaceae | *Cordia bicolor* | 1.68 | -1.83 | 8.98 | 1.72 | 1.96 | 0.00 | 3.68 |
|  |  | Burseraceae | *Protium tenuifolium* | 1.23 | -0.41 | 157.68 | 10.92 | 0.00 | 3.15 | 7.78 |
|  |  | Chrysobalanaceae | *Licania* sp1 [v. RLC-15703, tag. 678] | -1.46 | -0.34 | 6.08 | 0.42 | 0.00 | 0.00 | 0.42 |
|  |  | Ebenaceae | *Diospyros* sp1 [v. RLC-15612, tag. 23] | -0.43 | -1.21 | 0.00 | 0.00 | 0.00 | 0.00 | 0.00 |
|  |  | Euphorbiaceae | *Acalypha diversifolia* | 0.18 | 0.13 | 2.56 | 0.43 | 0.00 | 0.00 | 0.43 |
|  |  | Fabaceae | *Albizia* sp2 [v. RLC-15687, tag. 254] | 2.07 | -0.07 | 9.85 | 0.13 | 0.00 | 0.00 | 0.13 |
|  |  | Fabaceae | *Brownea ariza* | 0.90 | 0.62 | 23.06 | 0.00 | 0.00 | 0.00 | 0.00 |
|  |  | Fabaceae | *Cassia* sp [tag. 623] | -0.02 | -2.26 | 0.71 | 0.55 | 0.00 | 0.00 | 0.55 |
|  |  | Fabaceae | *Enterolobium* sp1 [v. RLC-15687, tag. 476] | 1.01 | -1.11 | 370.61 | 4.26 | 0.00 | 0.00 | 4.26 |
|  |  | Fabaceae | *Inga* sp1 [v. RLC-15590, tag. 73] | 0.83 | -0.44 | 693.54 | 58.36 | 3.34 | 12.71 | 48.99 |
|  |  | Fabaceae | *Inga* sp4 [v. RLC-15634, tag. 181] | 1.74 | -0.55 | 53.80 | 3.90 | 0.00 | 0.00 | 3.90 |
|  |  | Fabaceae | *Inga* sp6 [v. RLC-15589, tag. 87] | 1.64 | -1.06 | 2930.90 | 59.78 | 32.08 | 78.20 | 13.67 |
|  |  | Fabaceae | *Machaerium capote* | 1.13 | 0.10 | 2032.30 | 86.52 | 6.19 | 0.00 | 92.70 |
|  |  | Fabaceae | *Platymiscium pinnatum* | -0.27 | -1.63 | 0.00 | 0.00 | 0.00 | 0.00 | 0.00 |
|  |  | Fabaceae | *Senegalia* sp1 [v. RLC-15669, tag. 510] | 1.01 | -0.90 | 250.80 | 11.90 | 10.79 | 0.00 | 22.69 |
|  |  | Fabaceae | *Styphnolobium sporadicum* | 0.86 | -0.35 | 56.14 | 2.44 | 0.00 | 0.00 | 2.44 |
|  |  | Fabaceae | *Swartzia simplex* | 0.94 | 1.44 | 1128.24 | 0.00 | 0.00 | 0.00 | 0.00 |
|  |  | Fabaceae | *Swartzia* sp1 [v. RLC-15604, tag. 31] | 1.64 | 0.67 | 2486.80 | 35.44 | 0.00 | 0.00 | 35.44 |
|  |  | Lamiaceae | *Callicarpa acuminata* | -0.34 | -1.34 | 26.98 | 5.44 | 1.24 | 2.13 | 4.55 |
|  |  | Lauraceae | *Nectandra* sp [v. RLC-15618, tag. 116] | 0.66 | 0.03 | 242.35 | 16.87 | 7.01 | 6.65 | 17.23 |
|  |  | Lecythidaceae | *Gustavia hexapetala* | -1.02 | 0.20 | 490.78 | 14.84 | 0.00 | 16.96 | -2.12 |
|  |  | Lecythidaceae | *Gustavia superba* | 0.23 | -0.91 | 110.43 | 2.34 | 0.00 | 0.00 | 2.34 |
|  |  | Malvaceae | *Apeiba tibourbou* | 0.19 | -2.82 | 34.91 | 14.40 | 0.00 | 1.35 | 13.04 |
|  |  | Malvaceae | *Guazuma ulmifolia* | -0.40 | -1.43 | 348.90 | 2.06 | 0.00 | 0.00 | 2.06 |
|  |  | Malvaceae | *Hampea thespesioides* | 0.36 | -2.20 | 65.79 | 18.52 | 4.08 | 4.32 | 18.27 |
|  |  | Malvaceae | *Herrania laciniifolia* | 0.08 | -1.49 | 0.00 | 0.00 | 0.00 | 0.00 | 0.00 |
|  |  | Malvaceae | *Ochroma pyramidale* | 0.02 | -2.94 | 1441.24 | 72.10 | 0.00 | 42.79 | 29.31 |
|  |  | Malvaceae | *Pachira quinata* | 0.83 | -0.13 | 1414.20 | 3.67 | 0.19 | 54.15 | -50.30 |
|  |  | Malvaceae | *Pseudobombax septenatum* | 0.07 | -2.83 | 4.70 | 0.94 | 1.06 | 0.00 | 2.00 |
|  |  | Meliaceae | *Guarea* sp1 [v. RLC-15645, tag. 230] | 1.37 | 0.80 | 30.82 | 1.26 | 0.32 | 0.00 | 1.58 |
|  |  | Meliaceae | *Trichilia hirta* | 0.06 | -1.47 | 576.64 | 14.65 | 0.00 | 0.00 | 14.65 |
|  |  | Moraceae | *Brosimum alicastrum* | 0.50 | -0.37 | 681.37 | 691.79 | 0.00 | 0.00 | 691.79 |
|  |  | Moraceae | *Ficus* sp [tag. 24] | -0.12 | -0.15 | 0.00 | 0.00 | 0.00 | 0.00 | 0.00 |
|  |  | Moraceae | *Helianthostylis sprucei* | 0.48 | 0.34 | 2872.28 | 52.11 | 0.16 | 46.12 | 6.15 |
|  |  | Moraceae | *Maclura tinctoria* | -0.31 | -1.92 | 7.93 | 1.38 | 0.35 | 0.00 | 1.74 |
|  |  | Moraceae | *Sorocea* sp [v. RLC-15598, tag. 39] | 1.47 | 0.35 | 372.33 | 4.19 | 0.33 | 6.11 | -1.59 |
|  |  | Myrtaceae | *Eugenia* sp3 [v. JAC-3196, tag. 479] | -0.47 | 0.35 | 467.60 | 17.91 | 0.00 | 0.00 | 17.91 |
|  |  | Myrtaceae | *Eugenia* sp4 [v. RLC-15644, tag. 217] | 0.54 | 0.57 | 729.97 | 5.53 | 0.65 | 0.00 | 6.17 |
|  |  | Nyctaginaceae | *Neea macrophylla* | -0.15 | -2.37 | 192.32 | 7.48 | 0.00 | 10.23 | -2.75 |
|  |  | Piperaceae | *Piper* sp6 [v. RLC-15584, tag. 1] | -0.07 | -0.79 | 443.93 | 28.87 | 36.04 | 23.05 | 41.86 |
|  |  | Polygonaceae | *Coccoloba obovata* | 0.41 | 0.02 | 2.18 | 0.59 | 1.23 | 0.00 | 1.82 |
|  |  | Polygonaceae | *Ruprechtia* sp1 [tag. 768] | -0.18 | 1.33 | 9.33 | 1.47 | 0.00 | 0.00 | 1.47 |
|  |  | Polygonaceae | *Triplaris melaenodendron* | 0.89 | -0.32 | 391.87 | 40.92 | 0.00 | 7.96 | 32.96 |
|  |  | Rubiaceae | *Alseis blackiana* | -0.80 | -0.69 | 3140.66 | 100.95 | 4.55 | 0.00 | 105.50 |
|  |  | Rubiaceae | *Ixora* sp [v. RLC-15721, tag. 899] | -0.42 | -0.49 | 186.04 | 5.17 | 7.94 | 0.00 | 13.11 |
|  |  | Rubiaceae | *Ladenbergia* sp [v. RLC-15578, tag. 47] | -0.56 | -1.27 | 1090.69 | 2.56 | 0.00 | 0.00 | 2.56 |
|  |  | Rubiaceae | *Randia dioica* | -0.93 | -0.03 | 1451.78 | 26.92 | 1.74 | 7.69 | 20.97 |
|  |  | Rubiaceae | *Randia* sp [v. RLC-15620, tag. 99] | -0.44 | -0.70 | 2.32 | 0.38 | 0.00 | 0.00 | 0.38 |
|  |  | Rubiaceae | *Simira cordifolia* | -0.77 | -0.24 | 1122.33 | 29.12 | 0.27 | 0.00 | 29.39 |
|  |  | Rutaceae | *Zanthoxylum rhoifolium* | 0.16 | -1.40 | 0.56 | 0.09 | 0.00 | 0.00 | 0.09 |
|  |  | Salicaceae | *Banara ibaguensis* | -1.11 | 0.14 | 16.33 | 0.34 | 0.00 | 0.00 | 0.34 |
|  |  | Salicaceae | *Casearia aculeata* | -0.83 | -0.40 | 6.74 | 0.18 | 0.00 | 0.00 | 0.18 |
|  |  | Salicaceae | *Casearia praecox* | -1.02 | -0.18 | 124.59 | 13.41 | 21.42 | 4.99 | 29.84 |
|  |  | Sapindaceae | *Allophylus nitidulus* | 0.33 | 0.03 | 19.94 | 0.65 | 0.00 | 2.22 | -1.57 |
|  |  | Sapindaceae | *Cupania cinerea* | -1.26 | -0.10 | 0.98 | 0.12 | 0.00 | 0.00 | 0.12 |
|  |  | Sapindaceae | *Dilodendron costaricense* | 0.68 | -0.36 | 1585.88 | 6.30 | 0.04 | 65.79 | -59.44 |
|  |  | Sapindaceae | *Matayba* sp1 [v. RLC-15587, tag. 10] | 0.76 | 0.57 | 2168.33 | 17.56 | 0.12 | 0.00 | 17.68 |
|  |  | Sapotaceae | *Pouteria* sp5 [v. RLC-15728, tag. 770] | 0.28 | 0.50 | 94.72 | 17.07 | 0.00 | 1.53 | 15.54 |
|  |  | Solanaceae | *Solanum lepidotum* | -0.73 | -1.29 | 1.15 | 0.00 | 0.00 | 0.47 | -0.47 |
|  |  | Ulmaceae | *Ampelocera* sp1 [v. JAC-3203, tag. 865] | 1.14 | 0.88 | 41.44 | 3.37 | 1.06 | 0.00 | 4.43 |
|  |  | Urticaceae | *Cecropia peltata* | 0.20 | -2.62 | 1640.81 | 48.66 | 0.00 | 77.06 | -28.40 |
|  |  | Urticaceae | *Myriocarpa stipitata* | -0.42 | -2.66 | 288.93 | 7.62 | 0.00 | 8.64 | -1.02 |
|  |  | Urticaceae | *Urera caracasana* | -0.62 | -3.33 | 791.45 | 17.34 | 16.62 | 78.32 | -44.36 |
|  |  | Violaceae | *Rinorea* sp1 [v. JAC-3197, tag. 843] | -1.44 | 0.13 | 8.14 | 0.57 | 0.00 | 0.00 | 0.57 |
|  | **Taminango Research Station** | | | | |  |  |  |  |  |
|  |  | Bignoniaceae | *Handroanthus chrysanthus* | 0.66 | 0.87 | 17265.44 | 1115.92 | 16.42 | 210.68 | 921.66 |
|  |  | Burseraceae | *Bursera tomentosa* | 0.55 | -1.39 | 65.58 | 1.15 | 0.40 | 3.98 | -2.42 |
|  |  | Capparaceae | *Cynophalla flexuosa* | -0.45 | 0.68 | 640.83 | 14.77 | 0.00 | 9.69 | 5.08 |
|  |  | Erythroxylaceae | *Erythroxylum jaimei* | -0.95 | 0.76 | 16.35 | 0.06 | 0.00 | 0.00 | 0.06 |
|  |  | Euphorbiaceae | *Jatropha gossypiifolia* | -0.22 | -3.22 | 4.19 | 0.65 | 0.67 | 0.34 | 0.98 |
|  |  | Fabaceae | *Caesalpinia cassioides* | 0.27 | 0.45 | 135.86 | 1.92 | 2.13 | 3.21 | 0.85 |
|  |  | Fabaceae | *Vachellia pennatula* | 1.65 | 0.33 | 153.92 | 5.65 | 0.24 | 6.25 | -0.36 |
|  |  | Rutaceae | *Zanthoxylum fagara* | -0.72 | 0.73 | 1562.63 | 41.00 | 0.36 | 13.27 | 28.09 |
|  |  | Verbenaceae | *Lippia origanoides* | -0.33 | 1.09 | 21.01 | 0.26 | 0.00 | 1.97 | -1.71 |
|  | **El Vinculo Regional Park** | | | | |  |  |  |  |  |
|  |  | Achatocarpaceae | *Achatocarpus nigricans* | -0.41 | -0.16 | 1552.08 | 64.76 | 2.33 | 18.92 | 48.16 |
|  |  | Anacardiaceae | *Anacardium excelsum* | 1.44 | -1.08 | 2794.51 | 13.44 | 0.00 | 0.00 | 13.44 |
|  |  | Annonaceae | *Annona muricata* | 1.56 | -0.84 | 16.65 | 0.31 | 0.00 | 0.00 | 0.31 |
|  |  | Asteraceae | *Verbesina* sp [v. VLL-224, tag. D67] | -0.43 | -3.68 | 6.38 | 0.00 | 0.26 | 1.49 | -1.23 |
|  |  | Capparaceae | *Cynophalla amplissima* | 0.33 | 0.14 | 3123.39 | 92.40 | 0.00 | 28.23 | 64.17 |
|  |  | Erythroxylaceae | *Erythroxylum ulei* | -0.73 | 0.29 | 25.89 | 0.98 | 0.42 | 0.00 | 1.39 |
|  |  | Euphorbiaceae | *Croton gossypiifolius* | 1.17 | 0.03 | 56.12 | 0.25 | 2.03 | 13.77 | -11.49 |
|  |  | Euphorbiaceae | *Euphorbia cotinifolia* | -0.78 | -3.36 | 86.64 | 0.48 | 0.00 | 7.47 | -6.99 |
|  |  | Fabaceae | *Enterolobium* sp1 [v. VLL-228, tag. E24] | 0.09 | -2.19 | 3442.38 | 20.16 | 0.00 | 0.00 | 20.16 |
|  |  | Fabaceae | *Gliricidia sepium* | 0.91 | -1.58 | 714.11 | 22.47 | 0.00 | 0.00 | 22.47 |
|  |  | Fabaceae | *Machaerium capote* | 0.74 | 0.23 | 4513.18 | 200.13 | 0.11 | 4.08 | 196.16 |
|  |  | Fabaceae | *Pithecellobium dulce* | 0.40 | -1.97 | 68.54 | 2.34 | 0.00 | 0.00 | 2.34 |
|  |  | Fabaceae | *Pithecellobium lanceolatum* | 1.11 | -0.24 | 4259.99 | 108.38 | 0.43 | 62.22 | 46.59 |
|  |  | Fabaceae | *Pseudosamanea guachapele* | 1.58 | -0.10 | 28.99 | 3.20 | 0.00 | 0.00 | 3.20 |
|  |  | Fabaceae | *Senna spectabilis* | 1.81 | -1.22 | 37.33 | 0.20 | 0.00 | 0.00 | 0.20 |
|  |  | Fabaceae | *Vachellia farnesiana* | -0.13 | 1.63 | 15.41 | 0.66 | 0.00 | 0.00 | 0.66 |
|  |  | Lauraceae | *Ocotea veraguensis* | 0.36 | 0.21 | 7704.26 | 359.17 | 2.01 | 3.64 | 357.54 |
|  |  | Malpighiaceae | *Bunchosia pseudonitida* | -0.93 | 0.68 | 260.20 | 14.04 | 0.82 | 2.90 | 11.96 |
|  |  | Malpighiaceae | *Malpighia glabra* | -0.60 | -0.43 | 105.17 | 4.94 | 0.00 | 0.00 | 4.94 |
|  |  | Malvaceae | *Ceiba pentandra* | 0.21 | -1.54 | 199.03 | 11.11 | 0.00 | 0.00 | 11.11 |
|  |  | Malvaceae | *Guazuma ulmifolia* | 0.19 | -1.50 | 2967.65 | 40.96 | 0.00 | 37.52 | 3.44 |
|  |  | Meliaceae | *Trichilia pallida* | -0.81 | -0.71 | 27.81 | 1.38 | 0.09 | 0.00 | 1.47 |
|  |  | Moraceae | *Brosimum alicastrum* | 0.88 | 0.19 | 2154.56 | 134.82 | 3.70 | 1.31 | 137.21 |
|  |  | Moraceae | *Ficus zarzalensis* | -0.21 | -0.67 | 7.75 | 0.72 | 0.00 | 0.00 | 0.72 |
|  |  | Moraceae | *Sorocea trophoides* | 0.52 | 0.11 | 1136.64 | 35.97 | 3.46 | 7.50 | 31.93 |
|  |  | Myrtaceae | *Eugenia monticola* | -0.25 | 0.84 | 1100.86 | 53.69 | 6.10 | 51.80 | 7.99 |
|  |  | Myrtaceae | *Eugenia procera* | -0.79 | 0.86 | 10038.93 | 586.20 | 47.36 | 32.98 | 600.58 |
|  |  | Myrtaceae | *Psidium guineense* | -0.77 | 0.34 | 1.09 | 0.00 | 0.00 | 0.00 | 0.00 |
|  |  | Nyctaginaceae | *Guapira* sp1 [v. VLL-187, tag. O129] | -0.26 | -0.43 | 2704.48 | 100.50 | 5.00 | 29.35 | 76.15 |
|  |  | Piperaceae | *Piper amalago* | -0.83 | -0.73 | 2.47 | 0.05 | 0.00 | 0.00 | 0.05 |
|  |  | Rubiaceae | *Chiococca alba* | -0.16 | -0.53 | 422.31 | 9.17 | 1.11 | 0.58 | 9.69 |
|  |  | Rubiaceae | *Coffea arabica* | -0.87 | 0.31 | 2.94 | 0.03 | 0.10 | 0.00 | 0.13 |
|  |  | Rubiaceae | *Genipa americana* | 0.12 | -0.19 | 346.39 | 30.92 | 0.11 | 7.12 | 23.91 |
|  |  | Rutaceae | *Amyris pinnata* | -0.61 | 0.12 | 2143.52 | 19.79 | 0.77 | 100.86 | -80.31 |
|  |  | Rutaceae | *Zanthoxylum fagara* | -0.29 | 0.57 | 6.27 | 0.48 | 0.00 | 0.00 | 0.48 |
|  |  | Rutaceae | *Zanthoxylum rhoifolium* | -0.14 | -1.07 | 182.77 | 5.06 | 0.00 | 0.00 | 5.06 |
|  |  | Rutaceae | *Zanthoxylum schreberi* | -0.61 | -0.37 | 2987.50 | 130.46 | 7.88 | 20.89 | 117.45 |
|  |  | Rutaceae | *Zanthoxylum verrucosum* | -0.26 | -1.18 | 1199.99 | 58.49 | 1.85 | 0.00 | 60.34 |
|  |  | Salicaceae | *Casearia aculeata* | -1.37 | 0.01 | 297.68 | 15.83 | 0.07 | 3.08 | 12.81 |
|  |  | Salicaceae | *Xylosma intermedia* | -1.43 | -0.19 | 67.91 | 4.29 | 0.00 | 1.68 | 2.61 |
|  |  | Sapindaceae | *Cupania* sp1 [v. VLL-230, tag. E72] | 0.27 | 0.00 | 3107.66 | 56.90 | 0.00 | 86.01 | -29.11 |
|  |  | Sapindaceae | *Sapindus saponaria* | 0.98 | -0.28 | 1481.37 | 6.03 | 0.00 | 33.45 | -27.43 |
|  |  | Thymelaeaceae | *Daphnopsis* sp [v. VLL-243, tag. L150] | -0.18 | -0.71 | 196.68 | 7.66 | 0.00 | 0.00 | 7.66 |
|  |  | Urticaceae | *Urera simplex* | -0.38 | -3.44 | 21.71 | 0.33 | 0.23 | 2.44 | -1.88 |
|  |  | Verbenaceae | *Citharexylum kunthianum* | 0.56 | -0.11 | 373.58 | 5.70 | 0.29 | 26.38 | -20.39 |
| **Dry Savannas** | | | | | |  |  |  |  |  |
|  | **Tuparro National Park** | | | | |  |  |  |  |  |
|  |  | Achariaceae | *Lindackeria paludosa* | -0.51 | -0.14 | 74.64 | 10.24 | 0.00 | 0.00 | 10.24 |
|  |  | Achariaceae | *Mayna odorata* | -1.27 | -0.79 | 1.68 | 0.08 | 0.03 | 0.38 | -0.27 |
|  |  | Anacardiaceae | *Astronium graveolens* | 0.95 | -0.38 | 130.31 | 8.33 | 0.00 | 0.19 | 8.14 |
|  |  | Anacardiaceae | *Spondias mombin* | 0.68 | -0.83 | 12.33 | 0.54 | 0.00 | 0.00 | 0.54 |
|  |  | Annonaceae | *Duguetia odorata* | -0.26 | 0.29 | 115.85 | 6.99 | 0.00 | 0.00 | 6.99 |
|  |  | Annonaceae | *Guatteria metensis* | 1.56 | 0.48 | 20.99 | 0.37 | 0.00 | 1.36 | -0.99 |
|  |  | Apocynaceae | *Himatanthus articulatus* | -0.05 | -1.20 | 1867.66 | 49.40 | 1.44 | 24.62 | 26.22 |
|  |  | Bignoniaceae | *Handroanthus barbatus* | 1.56 | 1.00 | 693.35 | 5.75 | 0.00 | 26.94 | -21.19 |
|  |  | Bixaceae | *Cochlospermum orinocense* | 1.75 | -2.31 | 262.20 | 6.44 | 0.00 | 0.00 | 6.44 |
|  |  | Burseraceae | *Bursera simaruba* | 0.74 | -1.60 | 382.90 | 7.79 | 0.00 | 1.53 | 6.25 |
|  |  | Burseraceae | *Protium guianense* | 0.51 | 0.28 | 10057.62 | 271.40 | 0.26 | 0.00 | 271.66 |
|  |  | Capparaceae | *Capparidastrum sola* | -0.99 | 0.25 | 10.10 | 0.19 | 0.00 | 0.00 | 0.19 |
|  |  | Chrysobalanaceae | *Hirtella racemosa* | 0.92 | 1.11 | 68.18 | 2.17 | 0.00 | 0.00 | 2.17 |
|  |  | Chrysobalanaceae | *Licania apetala* | 0.74 | 0.94 | 531.98 | 16.13 | 0.00 | 0.00 | 16.13 |
|  |  | Chrysobalanaceae | *Licania micrantha* | 1.32 | 1.20 | 7600.18 | 192.13 | 0.00 | 0.54 | 191.59 |
|  |  | Chrysobalanaceae | *Licania parvifructa* | 1.62 | 1.22 | 399.74 | 4.98 | 0.00 | 0.00 | 4.98 |
|  |  | Chrysobalanaceae | *Licania* sp [v. RG-2387, tag. 116] | 2.58 | 0.74 | 154.17 | 4.45 | 0.00 | 0.00 | 4.45 |
|  |  | Chrysobalanaceae | *Licania* sp2 [v. RG-2367, tag. 35] | 0.80 | 0.82 | 712.47 | 12.98 | 0.00 | 0.00 | 12.98 |
|  |  | Clusiaceae | *Clusia umbellata* | 0.72 | 1.06 | 31.49 | 0.81 | 0.00 | 0.00 | 0.81 |
|  |  | Combretaceae | *Terminalia amazonia* | 1.34 | 0.19 | 594.60 | 30.11 | 0.00 | 0.00 | 30.11 |
|  |  | Connaraceae | *Connarus ruber* | 0.48 | 0.46 | 31.85 | 1.14 | 0.00 | 0.00 | 1.14 |
|  |  | Erythroxylaceae | *Erythroxylum macrophyllum* | -0.06 | 0.87 | 415.09 | 15.66 | 0.00 | 0.00 | 15.66 |
|  |  | Euphorbiaceae | *Mabea trianae* | 0.20 | -0.22 | 33.16 | 0.93 | 0.00 | 0.00 | 0.93 |
|  |  | Euphorbiaceae | *Sapium glandulosum* | 1.51 | -1.53 | 2290.53 | 55.65 | 0.00 | 22.72 | 32.93 |
|  |  | Fabaceae | *Clathrotropis macrocarpa* | 1.27 | -0.05 | 301.34 | 29.14 | 0.00 | 2.41 | 26.72 |
|  |  | Fabaceae | *Enterolobium schomburgkii* | 1.53 | 0.06 | 765.43 | 8.70 | 0.00 | 0.58 | 8.12 |
|  |  | Fabaceae | *Inga gracilifolia* | 1.48 | 1.26 | 3107.58 | 171.98 | 0.05 | 43.69 | 128.33 |
|  |  | Fabaceae | *Inga laurina* | 1.00 | 1.20 | 235.64 | 0.96 | 0.00 | 0.00 | 0.96 |
|  |  | Fabaceae | *Inga* sp [v. RG-2433, tag. 692] | 1.03 | 1.21 | 1800.61 | 113.10 | 0.00 | 51.81 | 61.29 |
|  |  | Fabaceae | *Machaerium biovulatum* | -0.01 | -2.58 | 114.22 | 3.11 | 0.00 | 0.00 | 3.11 |
|  |  | Fabaceae | *Pterocarpus* sp4 [v. RG-2420, tag. 389] | 1.12 | 0.02 | 695.79 | 10.18 | 0.00 | 0.00 | 10.18 |
|  |  | Fabaceae | *Tachigali guianensis* | 1.88 | 0.18 | 442.68 | 86.53 | 0.00 | 0.00 | 86.53 |
|  |  | Indet | Morf sp4 [v. RG-2430a, tag. 107] | 0.74 | -1.50 | 6063.79 | 132.92 | 0.00 | 0.00 | 132.92 |
|  |  | Lamiaceae | *Vitex orinocensis* | -0.06 | 0.62 | 322.24 | 3.97 | 0.00 | 0.00 | 3.97 |
|  |  | Lauraceae | *Ocotea schomburgkiana* | 0.70 | 0.53 | 216.84 | 12.51 | 0.38 | 2.65 | 10.25 |
|  |  | Lecythidaceae | *Eschweilera tenuifolia* | 1.17 | 1.19 | 8005.90 | 284.92 | 0.00 | 49.02 | 235.90 |
|  |  | Lecythidaceae | *Gustavia augusta* | -0.31 | -0.67 | 2370.81 | 43.24 | 0.29 | 10.54 | 33.00 |
|  |  | Lecythidaceae | *Lecythis chartacea* | 0.87 | 0.06 | 468.58 | 10.39 | 0.61 | 0.00 | 11.00 |
|  |  | Malvaceae | *Apeiba tibourbou* | 0.90 | -0.31 | 2067.01 | 28.11 | 0.00 | 0.00 | 28.11 |
|  |  | Malvaceae | *Pachira nukakica* | 2.21 | -2.43 | 4015.82 | 119.42 | 0.00 | 0.00 | 119.42 |
|  |  | Melastomataceae | *Graffenrieda rotundifolia* | 0.88 | -1.42 | 47.52 | 3.40 | 0.00 | 1.50 | 1.91 |
|  |  | Melastomataceae | *Miconia splendens* | -0.86 | -0.22 | 10.64 | 0.63 | 1.48 | 0.00 | 2.12 |
|  |  | Meliaceae | *Guarea glabra* | -0.36 | 0.05 | 578.66 | 23.83 | 0.00 | 2.37 | 21.46 |
|  |  | Moraceae | *Brosimum guianense* | 0.79 | 0.28 | 504.68 | 9.00 | 0.00 | 0.00 | 9.00 |
|  |  | Moraceae | *Clarisia racemosa* | 1.88 | 0.33 | 5.69 | 0.81 | 0.00 | 0.00 | 0.81 |
|  |  | Moraceae | *Ficus americana* | 1.97 | -0.94 | 3569.14 | 84.94 | 0.00 | 4.40 | 80.54 |
|  |  | Moraceae | *Ficus* sp [v. RG-2396, tag. 186] | 1.67 | -1.15 | 1.22 | 0.37 | 0.00 | 0.00 | 0.37 |
|  |  | Moraceae | *Ficus* sp1 [tag. 495] | 2.16 | -0.88 | 725.85 | 0.00 | 0.00 | 0.00 | 0.00 |
|  |  | Moraceae | *Ficus trigona* | 2.30 | 0.81 | 734.31 | 0.00 | 0.00 | 0.00 | 0.00 |
|  |  | Moraceae | *Pseudolmedia* sp1 [v. RG-2361, tag. 189] | 0.93 | 0.31 | 399.61 | 9.22 | 0.00 | 1.81 | 7.41 |
|  |  | Moraceae | *Sorocea muriculata* | 0.75 | 0.20 | 6.18 | 0.21 | 0.00 | 0.00 | 0.21 |
|  |  | Myrtaceae | *Calyptranthes multiflora* | 0.83 | -0.57 | 4.19 | 0.00 | 0.00 | 1.05 | -1.05 |
|  |  | Myrtaceae | *Eugenia florida* | 0.84 | 0.67 | 172.88 | 1.75 | 0.00 | 0.00 | 1.75 |
|  |  | Myrtaceae | Morf sp2 [v. RG-2372, tag. 47] | -1.58 | 0.05 | 227.82 | 7.49 | 0.00 | 0.00 | 7.49 |
|  |  | Myrtaceae | Morf sp3 [v. RG-2458, tag. ] | -0.63 | 0.75 | 320.37 | 9.47 | 0.00 | 0.00 | 9.47 |
|  |  | Myrtaceae | *Myrcia* sp1 [v. RG-2378, tag. 69] | -0.52 | 0.66 | 1306.41 | 59.27 | 0.29 | 0.37 | 59.19 |
|  |  | Myrtaceae | *Myrcia* sp2 [v. RG-2416, tag. 341] | -0.03 | 0.82 | 2.71 | 0.40 | 0.00 | 0.31 | 0.09 |
|  |  | Nyctaginaceae | *Neea ignicola* | -0.96 | 0.28 | 5.27 | 0.19 | 0.00 | 0.00 | 0.19 |
|  |  | Ochnaceae | *Ouratea* sp [v. RG-2380, tag. 83] | -0.01 | 0.91 | 67.03 | 5.14 | 0.10 | 0.00 | 5.23 |
|  |  | Olacaceae | *Heisteria acuminata* | 1.30 | 1.15 | 1107.08 | 32.93 | 0.00 | 21.44 | 11.49 |
|  |  | Phyllanthaceae | *Amanoa guianensis* | 1.63 | 1.67 | 408.97 | 13.12 | 0.00 | 0.00 | 13.12 |
|  |  | Rubiaceae | *Amaioua corymbosa* | -0.36 | -0.15 | 109.98 | 2.96 | 0.00 | 2.56 | 0.41 |
|  |  | Rubiaceae | *Cordiera myrciifolia* | -0.45 | 0.85 | 11.70 | 0.57 | 0.09 | 0.00 | 0.66 |
|  |  | Rubiaceae | *Coussarea paniculata* | -1.04 | -0.74 | 8.54 | 0.33 | 0.00 | 0.00 | 0.33 |
|  |  | Rubiaceae | *Palicourea rigida* | -1.14 | -1.02 | 75.98 | 5.01 | 0.00 | 0.00 | 5.01 |
|  |  | Rubiaceae | *Rudgea crassiloba* | -1.50 | -0.53 | 123.27 | 9.28 | 1.24 | 0.00 | 10.52 |
|  |  | Rubiaceae | *Simira rubescens* | -0.67 | 0.39 | 229.34 | 3.17 | 0.09 | 0.00 | 3.26 |
|  |  | Sapindaceae | *Matayba* sp [v. RG-2382, tag. 117] | 0.26 | 0.99 | 2352.02 | 72.45 | 0.23 | 6.33 | 66.35 |
|  |  | Sapotaceae | *Elaeoluma* sp [v. RG-2392, tag. 163] | 0.29 | 0.51 | 82.88 | 6.64 | 0.00 | 0.00 | 6.64 |
|  |  | Sapotaceae | *Pouteria plicata* | -0.23 | 0.38 | 1095.64 | 22.98 | 0.00 | 2.29 | 20.68 |
|  |  | Sapotaceae | *Pouteria* sp4 [v. RG-2406, tag. 234] | -0.31 | 0.20 | 1275.92 | 59.13 | 0.08 | 0.00 | 59.21 |
|  |  | Siparunaceae | *Siparuna guianensis* | -0.17 | -0.11 | 185.49 | 14.78 | 0.04 | 6.55 | 8.27 |
|  |  | Urticaceae | *Cecropia peltata* | 2.63 | -1.40 | 23.70 | 4.51 | 0.00 | 1.24 | 3.27 |
|  |  | Verbenaceae | *Petrea* sp [v. RG-2371, tag. 44] | 0.13 | -0.76 | 482.12 | 16.81 | 0.29 | 0.00 | 17.10 |
|  |  | Violaceae | *Rinorea pubiflora* | -1.31 | 0.01 | 35.50 | 0.20 | 0.06 | 5.88 | -5.63 |
|  |  | Vochysiaceae | *Vochysia vismiifolia* | 0.19 | 0.18 | 60.27 | 3.77 | 0.00 | 0.42 | 3.35 |

**Figure S3.** Null models for Functional Dissimilarity (F_Diss_) between biomass growth and mortality TPD’s at thresholds 20%, 50%, and 99% of probability. Significant β_O_ (*P*<0.001) indicated that dissimilarity between paired TPD demographic dimensions was more significant than expected by chance (999 randomizations). Hydraulic safety (hs); hydraulic efficiency (he); investments in tissues (it).

**Figure S4.** Functional Richness (F_Ric_) between biomass growth and mortality TPD’s at thresholds 20%, 50%, and 99% of probability. Significant differences between paired frequency distributions indicated different F_Ric_ of contrasted TPD’s demographic dimensions (*P*<0.001, 999 randomizations). Biomass growth of survivors (BG_S_), biomass growth of recruits (BG_R_), and biomass mortality (BM).

**REFERENCES**

Carmona, C.P., de Bello, F., Mason, N.W.H. & Lepš, J. (2016). Traits without borders: integrating functional diversity across scales. *Trends Ecol. Evol.*, 31, 382–394.

Carmona, C.P., de Bello, F., Mason, N.W.H. & Lepš, J. (2019). Trait probability density (TPD): measuring functional diversity across scales based on TPD with R. *Ecology*, 100, 1–8.

Poorter, L., van der Sande, M.T., Arets, E.J.M.M., Ascarrunz, N., Enquist, B., Finegan, B., *et al.* (2017). Biodiversity and climate determine the functioning of Neotropical forests. *Glob. Ecol. Biogeogr.*, 26, 1423–1434.

Prado-Junior, J.A., Schiavini, I., Vale, V.S., Arantes, C.S., van der Sande, M.T., Lohbeck, M., *et al.* (2016). Conservative species drive biomass productivity in tropical dry forests. *J. Ecol.*, 104, 817–827.

Talbot, J., Lewis, S.L., Lopez-Gonzalez, G., Brienen, R.J.W., Monteagudo, A., Baker, T.R., *et al.* (2014). Methods to estimate aboveground wood productivity from long-term forest inventory plots. *For. Ecol. Manage.*, 320, 30–38.

Vicente-Serrano, S.M., Zouber, A., Lasanta, T. & Pueyo, Y. (2012). Dryness is accelerating degradation of vulnerable shrublands in semiarid mediterranean environments. *Ecol. Monogr.*, 82, 407–428.
